# Synthesis and Characterization of Tricarbonyl-Re/Tc(I) Chelate Probes Targeting the G Protein-Coupled Estrogen Receptor GPER/GPR30

**DOI:** 10.1371/journal.pone.0046861

**Published:** 2012-10-15

**Authors:** Ritwik Burai, Chinnasamy Ramesh, Tapan K. Nayak, Megan K. Dennis, Bj K. Bryant, Eric R. Prossnitz, Jeffrey B. Arterburn

**Affiliations:** 1 Department of Chemistry and Biochemistry, New Mexico State University, Las Cruces, New Mexico, United States of America; 2 Department of Cell Biology and Physiology, University of New Mexico Health Science Center, Albuquerque, New Mexico, United States of America; 3 University of New Mexico Cancer Center, University of New Mexico Health Science Center, Albuquerque, New Mexico, United States of America; University of Torino, Italy

## Abstract

The discovery of the G protein-coupled estrogen receptor GPER (also GPR30) and the resulting development of selective chemical probes have revealed new aspects of estrogen receptor biology. The potential clinical relevance of this receptor has been suggested from numerous studies that have identified GPER expression in breast, endometrial, ovarian and other cancers. Thus GPER can be considered a candidate biomarker and target for non-invasive imaging and therapy. We have designed and synthesized a series of organometallic tricarbonyl-rhenium complexes conjugated to a GPER-selective small molecule derived from tetrahydro-3H-cyclopenta[c]quinoline. The activity and selectivity of these chelates in GPER-mediated signaling pathways were evaluated. These results demonstrate that GPER targeting characteristics depend strongly on the structure of the chelate and linkage. Ethanone conjugates functioned as agonists, a 1,2,3-triazole spacer yielded an antagonist, and derivatives with increased steric volume exhibited decreased activities. Promising GPER selectivity was observed, as none of the complexes interacted with the nuclear estrogen receptors. Radiolabeling with technetium-99m in aqueous media was efficient and gave radioligands with high radiochemical yields and purity. These chelates have favorable physicochemical properties, show excellent stability in biologically relevant media, exhibit receptor specificity and are promising candidates for continuing development as diagnostic imaging agents targeting GPER expression in cancer.

## Introduction

Estrogens are involved in a diverse array of physiological responses. The genomic roles of the nuclear estrogen receptors ERα and ERβ have been characterized in greatest detail. ERα, and to some extent ERβ, are important drug targets because of their roles in development, reproduction, skeletal physiology and the nervous, cardiovascular, and immune systems. Estrogen also rapidly triggers a variety of secondary messenger (non-genomic) signaling events that contribute to complex physiological, morphological and behavioral effects. Estrogen-responsiveness constitutes a major determinant of therapy selection and prognosis in breast cancer with both genomic and non-genomic pathways regulating tumor biology [1]–[4]. The recent identification of the involvement of a G protein-coupled estrogen receptor GPR30 (IUPHAR designation: GPER) in tumor signaling pathways and studies demonstrating the prognostic value of assessing GPER expression suggest GPER may serve as a potentially important biomarker and therapeutic target in cancer [5]–[9].

**Figure 1:**
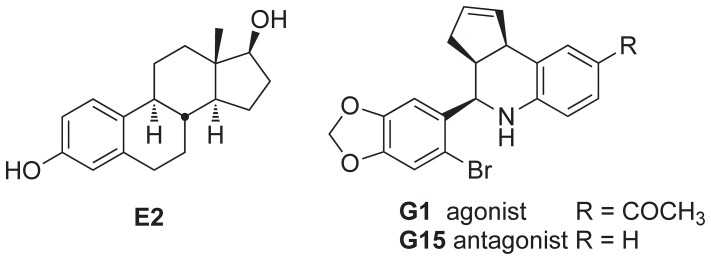
Structures of 17β-estradiol (E2), and GPER-selective agonist G-1, and antagonist G15.

We have developed the first GPER-selective agonist **G-1**, a tetrahydro-3*H*-cyclopenta[c]quinoline, and the structurally related antagonists **G15** and **G36**, all of which exhibit high affinity for GPER and high selectivity for GPER over ERα/β (Figure 1) [10]–[12]. Structure activity studies have identified the important role of the 6-bromo-benzo [1], [3]dioxolane substituent, and the presence of an ethanone hydrogen-bond acceptor group at the C8-position was associated with agonism. These functional probes have been used to distinguish GPER-mediated effects in a wide variety of *in vitro* and *in vivo* studies, particularly in systems expressing multiple estrogen receptors, generating high confidence in their use as leads in drug discovery programs [13]. Based on the demonstrated receptor selectivity of this scaffold, we initiated a program focusing on the development of novel targeted imaging agents for the *in vivo* characterization of GPER expression in normal and disease model systems.

**Figure 2:**
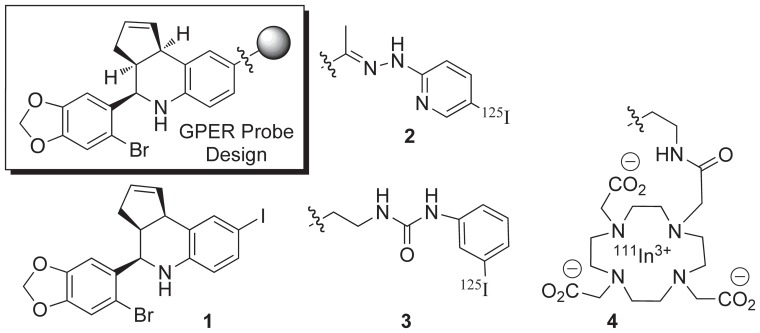
General design of tetrahydro-3*H*-cyclopenta[*c*]quinoline GPER-targeted imaging agents. Structures of first generation compounds: C8-iodo derivative **1**, ^125^I-labeled hydrazone derivative **2**, ^125^I-labeled urea derivative **3**, ^111^In-labeled DOTA chelate **4**.

We recently described the generation of the first radiolabeled agents based on the GPER-targeting tetrahydro-3H-cyclopenta[c]quinoline ( = “G”) scaffold of **G-1**/**G15**, incorporating the γ-emitting isotopes ^125^I, and ^111^In (Figure 2) [14], [15]. While the C8-iodo compound **1** exhibited promising GPER-targeting characteristics, radiolabeling of the stannane precursor resulted in poor yields and competing deiodination due to the strong electron-donating effect of nitrogen and prevented practical application of this agent. The pendant hydrazone **2** and urea **3** derivatives underwent ^125^I-radiolabeling and were effective competitive ligands for GPER binding, but showed poor tumor targeting characteristics using *in vivo* xenograft model studies. The relatively high background and non-target tissue uptake was attributed to the lipophilicity of the pendant groups and complications due to rapid metabolism. In complementary studies, we constructed a series of acyclic and macrocyclic polyamino-polycarboxylate ligands and evaluated the resulting ^111/113^In(III) chelates to determine the effect of ionic charge on GPER targeting *in vitro*. The neutral DOTA conjugate **4** was capable of activating rapid GPER-mediated signaling pathways while charged complexes were inactive. Rapid initiation of GPER-mediated signaling by neutral, but not charged, synthetic estrogen probes has also been demonstrated [16]. These results are consistent with previous studies demonstrating the predominant intracellular localization for GPER in the endoplasmic reticulum of most cell types, with only minor amounts detectable at the plasma membrane [17]. Chelate **4** exhibited receptor-mediated uptake in the uterus, mammary glands, adrenal glands and tumor with the tumor being clearly visualized, although high uptake in the intestines, hepatobiliary excretion and the formation of polar metabolites limit the usefulness of this agent. These studies have identified the need for alternative radiolabeling modalities to improve intracellular targeting properties and obtain enhanced *in vivo* performance.

Improvements in small animal imaging scanners for single photon emission computed tomography (SPECT) have increased the resolution and sensitivity of detection affording numerous advantages for preclinical studies and opportunities for employing the favorable nuclear decay properties of the important diagnostic radionuclide ^99m^Tc (T_1/2_ = 6 h, 140 keV γ-radiation) that is widely available through ^99^Mo/^99m^Tc generators [18]. Whereas all isotopes of technetium are radioactive, the third-row congener rhenium (^185^Re and ^187^Re) serves as an effective non-radioactive chemical surrogate for technetium, yet is also available as the medically useful radioisotopes: ^186/188^Re. The organometallic M(CO)_3_^+^ core (M = ^99m^Tc, Re) is conveniently generated in aqueous media and offers favorable characteristics that are advantageous for the design of small molecule imaging agents, such as a small steric profile and promiscuous chelation chemistry with a variety of bidentate and tridentate ligands to yield kinetically stable complexes [19].

**Figure 3:**
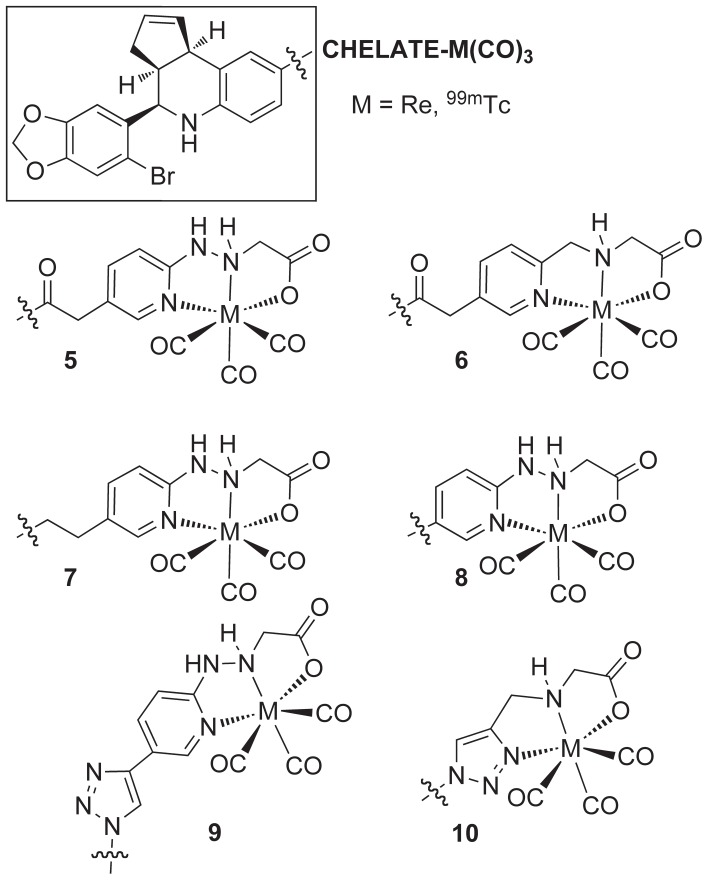
Structures of GPER-targeted organometallic M(CO)_3_^+^ chelates (M = Re, ^99m^Tc).

In this study, we describe the synthesis of a promising new class of GPER-targeted tricarbonyl-Re(I) chelates **5–10** (Figure 3), and profile their targeting properties, activity and selectivity using cell-based functional assays for receptor-mediated signaling. Within the series of Re-chelates evaluated, the connecting linkage and chelate structure were found to contribute significantly to the physicochemical and biological properties, effectively determining the functional role of the compounds as agonists or antagonists of GPER-mediated signaling. Efficient radiolabeling with [^99m^Tc(CO)_3_(H_2_O)_3_]^+^ provides a promising new class of GPER-targeted SPECT imaging agents.

## Results and Discussion

### Synthesis of chelate ligands

We designed a series of six new GPER-targeted organorhenium probes (**5–10**) using the tetrahydro-3*H*-cyclopenta[*c*]quinoline scaffold shared by **G-1** and **G15**. We have previously described an efficient, diastereoselective synthetic route for G-1 [20]. Three different chelating heterocyclic aminocarboxylate ligands with demonstrated capacity for the formation of neutral tricarbonylrhenium(I) complexes were selected; namely pyridin-2-yl-hydrazinylethanoic acid (**5**,**7**–**9**) [21], [22], pyridin-2-yl-methylaminoethanoic acid (**6**) [23], [24], and 1,2,3-triazol-4-yl-methylaminoethanoic acid (**10**) [25], [26]. The C8 position of the cyclopenta[c]quinoline scaffold was selected for attachment of the chelating ligands, based on our previous structure-activity studies demonstrating the plasticity of this site for derivatization. The proximal functionality and length of the connecting linkages between targeting moiety and chelate groups were varied within this series to evaluate the combined structural effects on the interaction of the complexes with GPER.

**Figure 4:**
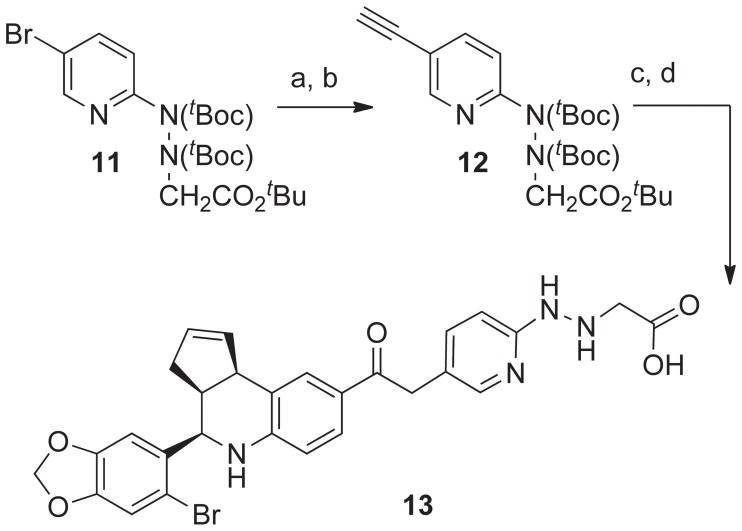
Synthesis of pyridin-2-yl-hydrazinylethanoic acid ligand 13. Reagents and conditions: a) TMS-acetylene, PdCl_2_(PPh_3_)_2_ (10 mol%), CuI (8 mol%), Hunigs base, NMP, 80°C, 2 h, 94%; b) AgOTf (20 mol%), CH_2_Cl_2_/MeOH/H_2_O (7∶4∶1), rt, 20 h, 80%; c) 1 (0.85 eq), PdCl_2_(PPh_3_)_2_ (10 mol%), CuI (10 mol%), Et_3_N, NMP, rt, 6 h, 79%; d) TFA/DCM (0.8∶1.0), 45 min, **13**, 96%.

The *^t^*Boc-protected 5-bromopyridin-2-yl-hydrazinylethanoate **11** was converted to the 5-ethynyl derivative **12** using a palladium-catalyzed Sonogashira coupling with trimethylsilylacetylene, followed by silver(I)triflate-mediated removal of the silyl group (Figure 4). A second Sonogashira coupling of alkyne **12** with the C8-iodo compound **1** gave the corresponding ethyne-linked synthetic intermediate. Removal of the *^t^*Boc- and *^t^*butyl-ester groups with trifluoroacetic acid was accompanied by regioselective hydrolysis of the alkyne to the ethanone group [27]. The pyridin-2-yl-hydrazinylethanoic acid ligand **13** was purified by silica gel chromatography.

**Figure 5:**
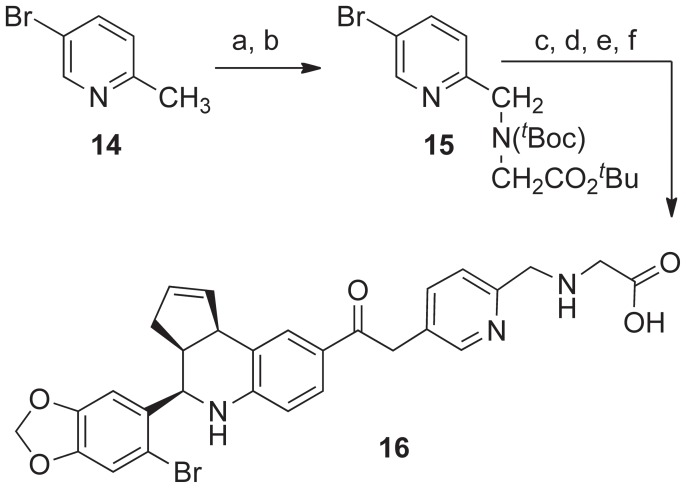
Synthesis of pyridin-2-yl-methylaminoethanoic acid ligand 16. Reagents and conditions: a) NBS (1eq), AIBN (20 mol%), CCl_4_, 65°C, 20 h, 43%; b) tert-butoxycarbonylamino-acetic acid tert-butyl ester (1 eq), NaH (1 eq), DMF, rt, 2 h, 9 83%; c) TMS-acetylene, PdCl_2_(PPh_3_)_2_ (10 mol%), CuI(8 mol%), Hunigs base, NMP, 80°C, 2 h, 98%; d) AgOTf (20 mol%), CH_2_Cl_2_/MeOH/H_2_O (7∶4∶1), rt, 20 h, 90%; e) 1 (0.85 eq), PdCl_2_(PPh_3_)_2_ (10 mol%), CuI( 10 mol%), Et_3_N, NMP, rt, 6 h, 90%; f) TFA/DCM (0.8∶1.0), 45 min, **16**, 70%.

Radical initiated bromination of 5-bromo-2-methylpyridine **14** with N-bromosuccinimide, followed by N-alkylation with *^t^*Boc-glycine-*^t^*butyl ester gave the picoline amine derivative **15** (Figure 5). A step-wise sequence of Pd-catalyzed ethynylation with trimethylsilylacetylene, removal of TMS-group, Sonogashira coupling of the resulting ethyne with the C8-iodo compound **1**, and finally deprotection of the *^t^*Boc- and *^t^*butyl-ester groups occurred with concomitant hydrolysis of the alkyne in TFA to afford the ethanone-linked pyridin-2-yl-methylaminoethanoic acid ligand **16**.

**Figure 6:**
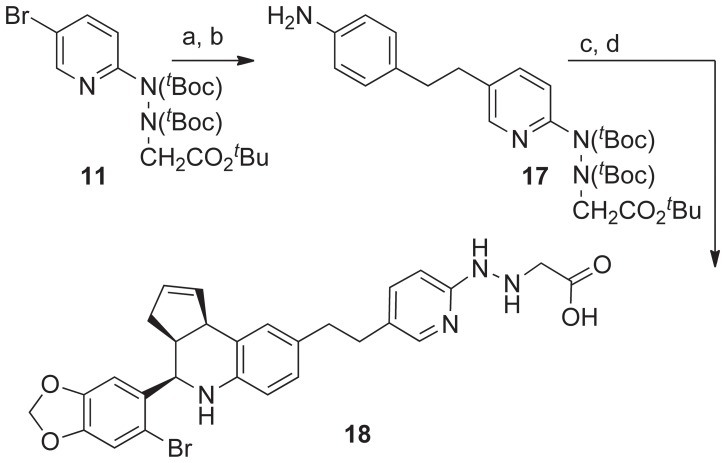
Synthesis of ethane-linked pyridin-2-yl-hydrazinylethanoic acid ligand 18. Reagents and conditions: a) 4-ethynylaniline (1.5 eq), Pd(OAc)_2_ (10 mol%), PPh_3_ (20 mol%), CuI (20 mol%), 55°C, 2 h, 68%; b) Pd/C (10%), H_2_(30 psi), EtOH, 3 h, 91%; c) 6-bromopiperonal (1 eq), KHSO_4_ (1 eq), cyclopentadiene (5 eq), rt, 24 h, 42%; d) TFA/DCM (0.8∶1.0), 45 min, **18**, 80%.

Facile palladium-catalyzed hydrodebromination of the bromo-benzodioxole group complicated direct attempts to prepare the ethane-bridged chelate ligand **18** by catalytic hydrogenation of the ethyne-linked synthetic intermediate produced in Figure 4. Therefore, we designed an alternative approach to **18** using the protected aniline derivative **17** as a constituent of the three-component aza-Diels Alder (Povarov) reaction (Figure 6). The palladium-catalyzed C-C coupling of 4-ethynylaniline with protected 5-bromopyridin-2yl-hydrazinylethanoic derivative **11**, followed by catalytic hydrogenation of the resulting alkyne using 10% Pd/C gave the aniline derivative **17**. The cyclization of **17** with 6-bromopiperonal and cyclopentadiene was catalyzed by KHSO_4_ to provide the tetrahydro-3*H*-cyclopenta[*c*]quinoline derivative **18** in moderate yield. The endo-diastereoselectivity achieved with this catalyst was relatively low (3.3∶1); however, attempts to improve this using the scandium catalyst Sc(OTf)_3_ were unsuccessful due to competing chelation by the hydrazine-2-yl pyridine. Removal of the *^t^*butyl-protecting groups using TFA, gave the ethane-linked pyridin-2-yl-hydrazinylethanoic acid ligand **18**.

**Figure 7:**
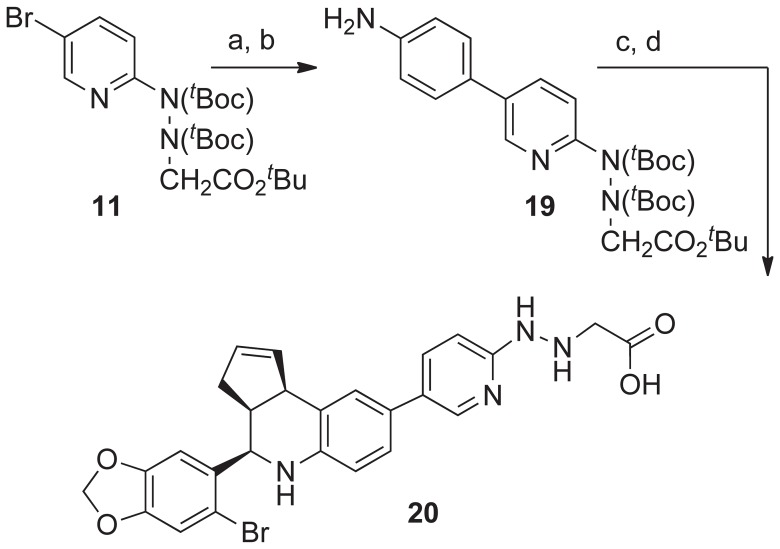
Synthesis of pyridin-2-yl-hydrazinylethanoic acid ligand 20. Reagents and conditions: a) **5**, bispinacolatodiboron (1.5 eq), PdCl_2_dppf*CH_2_Cl_2_ (8 mol%), KOAc (5 eq), DMF, 80°C, 16 h, 94%; b) 4-iodoaniline (0.9 eq), KOH (2 eq), and Pd-catalyst (4 mol %) [29], MeOH/H_2_O (3∶1), 60°C, 3 h, 13 70%; c) 6-bromopiperonal (1 eq), Sc(OTf)_3_ (10 mol%), cyclopentadiene (5 eq), MeCN, rt, 4 h, 74%; d) TFA/DCM (0.8∶1.0), 45 min, **20**, 90%.

The *^t^*Boc-protected 5-bromopyridin-2-yl-hydrazinylethanoate derivative **11** was converted to the boronic ester using PdCl_2_(dppf) and bis-pinacolatodiboron (Figure 7). The Suzuki coupling of this derivative with 4-iodoaniline using an oxime-palladacycle catalyst gave the protected aniline derivative **19** in good yield [28]. The KHSO_4_ catalyzed cyclization of **19** with 6-bromopiperonal and cyclopentadiene in acetonitrile gave the tetrahydro-3*H*-cyclopenta[*c*]quinoline derivative in good yield. Treatment with TFA gave the pyridin-2-yl-hydrazinylethanoic acid ligand **20**.

**Figure 8:**
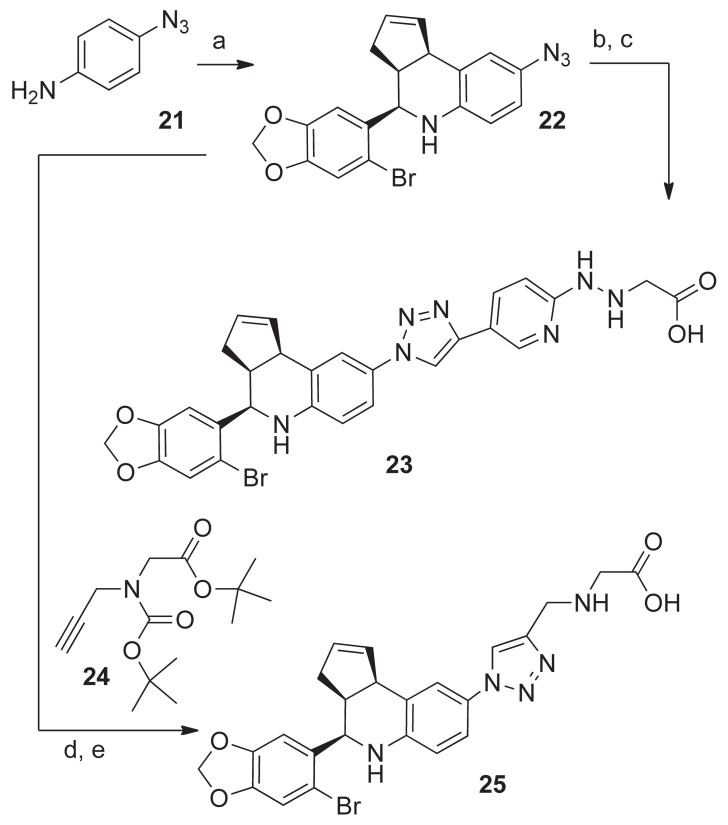
Synthesis of triazole ligands 23 and 25. Reagents and conditions: a) **21**, 6-bromopiperonal (1 eq), cyclopentadiene (5 eq), Sc(OTf)_3_ (10 mol%), rt, 4 h, **22** 90%; b) **12** (1 eq), CuSO_4_ 
5H_2_O (10 mol%), sodium ascorbate (20 mol%) H_2_O/*^t^*BuOH (1∶ 1), 60°C, 20 h, 52%; c) TFA/DCM (0.8∶1.0), 45 min, **23**, 96%; d) **22** (1eq), **24** (1 eq), CuSO_4_.5H_2_O (10 mol%), sodium ascorbate (20 mol%) H_2_O/*^t^*BuOH (1∶1), 60°C, 3 h, 60%; e) TFA/DCM (0.8∶1.0), 2.0 h, **25**, 75%.

The synthetic approaches used for the preparation of triazole ligands **23** and **25** were both based on the C8-azide intermediate **22** (Figure 8). The aza-Diels Alder cyclization of 4-azidoaniline **21** with 6-bromopiperonal and cyclopentadiene in acetonitrile was catalyzed by Sc(OTf)_3_ and gave **22** in high yield [29]. The Cu(II)-catalyzed “Click” reaction of **22** with the protected 5-ethynyl derivative **12** in aqueous *tert*-butanol gave the triazole-linked derivative in moderate yield. Deprotection with TFA, followed by silica gel chromatography yielded the triazole-linked pyridin-2-yl-hydrazinyl ligand **23**. The “click” to chelate approach was used for the coupling of **22** with the protected N-propargyl-glycine derivative **24** [25], [26]. Removal of the *^t^*butyl-groups with TFA gave the 1,2,3-triazol-4-yl-methylaminoethanoic acid ligand **25**.

### Complexation with [Re(CO)_3_(H_2_O)_3_]^+^

All of the aminocarboxylate conjugates evaluated were effective for complexation of Re(CO)_3_(H_2_O)_3_ in aqueous ethanol as shown in Table 1. The chelation occurred at ambient temperature; however, reduced reaction times and increased yields of the complexes were achieved at higher temperatures. Evaluation of the stability of the triazol-4-yl-methylamine, picolinamine and pyridin-2yl-hydrazine ligands in aqueous ethanol buffered with NaHCO_3_ at 90°C showed no observable decomposition after 1 h. The different solubilities of the ligands were considered when selecting the EtOH/H_2_O solvent mixture. The picoline-based chelate **6-Re** was more hydrophobic than the pyridylhydrazine complex **5-Re**, and while this may be expected from the isosteric replacement of “NH” with “CH_2_” groups, this substitution resulted in large differences in chromatographic mobility using reverse phase HPLC where the retention times were 20.63 min and 16.32 min respectively. The complexation of triazol-4-yl-methylamine ligand **25** at room temperature was relatively slow. Increasing the reaction temperature to 65°C for 1 h gave complex **10-Re** in high yield (92%).

**Table 1:** Complexation with Re(CO)_3_(H_2_O)_3_Cl.

Ligand	Time (h)	Temp	Complex	Yield (%)^a^
13	5	RT	5-Re	75
16	2.5	RT	6-Re	60
18	3	RT	7-Re	62
20	2	RT	8-Re	87
23	6	RT	9-Re	68
25	1	65°C	10-Re	92

a Isolated yields of pure compounds after silica gel chromatography.

### Ligand Interactions with GPER and ERα/β Receptors

The series of tricarbonylorganorhenium(I) complexes **5-Re : 10-Re** was evaluated using established functional assays for GPER and profiled for potential cross-reactivity via the nuclear estrogen receptor ERα (Table 2). The capacity of the complexes to elicit rapid mobilization of intracellular calcium was evaluated in SKBr3 breast cancer cells, which are ERα- and ERβ-negative but express GPER. The pyridylhydrazine and picolinamine complexes **5** and **6** were both potent agonists of GPER-mediated calcium mobilization. These complexes possess conserved ethanone groups in the connecting linkage, analogous to the parent agonist ligand **G-1**, further supporting structure-activity models that indicate a role for the hydrogen bond accepting properties of the ethonanone group in agonism of this ligand. In sharp contrast, complexes **7-Re**, **8-Re** and **10-Re** were inactive as agonists at concentrations up to 30 µM. The assay was then modified to assess possible antagonism by examining the capacity of the complexes to block Ca^2+^ mobilization by 17β-estradiol. The triazole-linked conjugate **9-Re** was identified as an antagonist of estrogen activation, while complexes **7-Re**, **8-Re** and **10-Re** had no effect on this response.

**Table 2:** Functional Assay Profile of GPER and ERα Activity.

Compound	GPER∶Ca^2+^ a	GPER∶PI3K^b^	ERα∶PI3K^b^
**5**	agonist	agonist	inactive
**6**	agonist	agonist	inactive
**7**	inactive	inactive	inactive
**8**	inactive	inactive	inactive
**9**	antagonist	antagonist	inactive
**10**	inactive	inactive	inactive

a Activity determined by the ability of compounds at 1 µM to induce calcium mobilization or of compounds at 10 µM to block calcium mobilization induced by 200 nM 17β-estradiol.

b Activity determined by the ability of compounds at 10 µM to induce nuclear translocation of PH-RFP or to block translocation induced by 100 nM 17β-estradiol.

The stimulation of GPER as well as ERα and ERβ by the native hormone 17β-estradiol activates PI3K and results in the nuclear accumulation of phosphatidylinositol-3,4,5-trisphosphate (PIP3), as shown by the nuclear translocation of an Akt PH domain fluorescent protein fusion protein reporter (PH-RFP). To determine whether these complexes activated the PI3K pathway, we employed COS7 cells transiently transfected with either GPER-GFP or ERα-GFP together with PH-RFP that serves as a reporter of PIP3 production. The complexes **5-Re** and **6-Re** were again found to be agonists of GPER-mediated PI3K activation, confirming their activity profile. Switching the assay protocol to detect antagonism of PI3K activation by 17β-estradiol, the complex **9-Re** was similarly identified as an antagonist. None of the complexes were able to activate PI3K through ERα, demonstrating that the conjugation of linked chelate groups did not alter the selectivity profile to allow interaction with the nuclear receptor, consistent with the observation that there was no significant binding to ERα or ERβ at concentrations up to 10 µM, where 17β-estradiol yields a K*_i_* of 0.3–0.5 nM (data not shown).

The activity profile in receptor-mediated signaling obtained from functional assays reveals the importance of structural effects associated with the linkage to the heterocyclic aminocarboxylate ligand at the C8 position of the tetrahydroquinoline scaffold. The pyridylhydrazine and picolinamine complexes **5-Re** and **6-Re** possess ethanone linkages that are analogous to the methyl ketone group of **G-1**, and were similarly found to be potent agonists of GPER signaling in both the calcium and PI3K assays. In contrast, the triazole-linked complex **9-Re** antagonized GPER-mediated signaling in both of these functional assays. The 1,2,3-triazole linkage is capable of functioning as a hydrogen-bond acceptor; however, the increased steric constraints and rigid planar ring structure in **9-Re** may prevent the required conformational alignment in the receptor-bound complex. The ethane-linked complex **7-Re** was inactive towards initiating or blocking GPER-mediated signaling. The conformational mobility of the flexible ethane linkage would produce a relatively large rotational steric volume and unfavorable entropic contribution that may impede the process of ligand-binding, accompanied by reduced affinity due to the hydrophobicity and absence of a H-bond accepting group in this linkage. The direct connection of heterocyclic chelates to the quinoline scaffold in compounds **8-Re** and **10-Re** contributes a relatively large steric volume in this region which precludes interactions with GPER. The extension of the linkage through a planar triazole group yielded antagonist complex **9**, and emphasizes the importance of the linkage structure on receptor targeting properties.

### Radiolabeling with [^99m^Tc(CO)_3_(H_2_O)_3_]^+^

The tricarbonyl approach was used to prepare the ^99m^Tc-radiolabeled complexes **5–8**. The [^99m^Tc(CO)_3_(H_2_O)_3_]^+^ intermediate was successfully prepared with a radiochemical purity of >95% (n>50). The resultant [^99m^Tc(CO)_3_(H_2_O)_3_]^+^ intermediate was mixed with the corresponding ligand and stirred at room temperature for 2 hours. These conditions resulted in over 95% incorporation of the [^99m^Tc(CO)_3_(H_2_O)_3_]^+^ into the pyridin-2-yl hydrazine **5-Tc** and picolylamine **6-Tc** complexes. A reduction to 85% incorporation was observed for the directly linked pyridin-2-yl hydrazine **8-Tc** complex. The radiolabeled complexes were conveniently purified by reverse-phase solid phase extraction, efficiently removing excess ligand and inorganics. In order to assess a more rapid radiosynthetic method for improved specific activity, the [^99m^Tc(CO)_3_(H_2_O)_3_]^+^ intermediate was mixed with the ligands and heated at 80°C for 30 min. Under these complexation conditions, degradation products were evident by HPLC analysis and the radiochemical purity was less than 70%. All ^99m^Tc-labeled complexes demonstrated good stability (>95%) in mouse plasma and PBS buffer after incubation at 37°C for 24 h. The complexes were stable in the presence of biologically relevant chelating ligands, exhibiting less than 10% transchelation upon incubation with 1 mM cysteine solution or 1 mM histidine solution at 37°C for 24 h. The log *P*_(o/w)_ values (mean ± SEM, n = 4) of the ethanone-linked pyridin-2-yl hydrazine complex **5-Tc** (4.6±0.3) was lower than that for the picolinamine complex **6-Tc** (5.0±0.1), revealing the contribution of the hydrazine group to the hydrophilicity of the complex. Increased log *P*_(o/w)_ values were also observed for the ethane-linked **7-Tc** (4.9±0.1) and directly connected pyridin-2-yl hydrazine complex **8-Tc** (5.5±0.1). Excessive lipophilicity was previously identified as a major limitation resulting in non-target tissue uptake for the urea- and hydrazone-linked I-125 radiolabeled analogs **2** and **3** with log *P*_(o/w)_ values of 7.00 and 6.35 respectively [13]. The tricarbonyl-Tc(I) chelate structure in **5-Tc** therefore represents a significant improvement in log *P*_(o/w)_ values compared to previous generations of GPER-targeted imaging agents.

### Conclusions

We have identified a new class of organometallic probes that selectively targets GPER in cellular assays. The tridentate heterocyclic aminocarboxylate ligands formed stable complexes from the cationic precursor [Re(CO)_3_(H_2_O)_3_]^+^ in aqueous ethanol. These conditions were successfully adapted for efficient ^99m^Tc-radiolabeling. The ethanone-linked complexes **5-Re** and **6-Re** yielded selective activation of GPER-mediated signaling pathways comparable to the parent ligand **G-1**, while the triazole-linked complex **9-Re** was an antagonist capable of blocking GPER signaling. These results demonstrate that structural features of the linkage and chelate were critical determinants of receptor-mediated signaling through GPER. We anticipate that continuing *in vivo* SPECT imaging studies of GPER expression with the promising new probe complex **5-Tc** using will reveal important new insights on the role of GPER in normal and disease states.

## Materials and Methods

Reagents and solvents were purchased from commercial sources and used without further purification. Preparative chromatography was performed using Sorbent technologies prepacked silica gel columns under medium pressure with ethyl acetate/hexanes (EtOAc/Hexanes) or methanol/dichloromethane (MeOH/CH_2_Cl_2_) as eluent. Reactions were followed by thin-layer chromatography (TLC) on silica gel (60 Å pore size, 5–17 µm) polyester backed sheets that were visualized under a UV lamp, iodine vapor, phosphomolybdic acid, or anisaldehyde. ^1^H NMR spectra were acquired at 300 MHz or 400 MHz, and ^13^C NMR were acquired at 75 MHz or 100 MHz spectrometers at ambient temperatures (18±2°C) unless otherwise noted. The ^1^H NMR spectra in CDCl_3_ were referenced to TMS unless otherwise noted. The ^13^C {1H} NMR spectra were recorded at 75 or 100 MHz and referenced relative to the ^13^C {1H} peaks of the solvent. Spectra are reported as (ppm), (multiplicity, coupling constants (Hz), and number of protons). Tri and Py are abbreviations of triazole and pyridine, respectively. Analytical HPLC was obtained using a Waters 2695 HPLC with Waters 2996 Photodiode Array (PDA) and Micromass ZQ ESI-MS detection (cone voltage 62 V, Capillary Voltage 3 kV). The compound (1 mg/mL CH_3_CN, 20 µL) was injected into Waters Symmetry® C_18_ 5 µm 3.0×150 mm column eluted with CH_3_CN/H_2_O as specified. High resolution mass spectra were obtained from the University of California at Riverside.

### General Procedure A; Deprotection of *^t^*Boc-ligands

Trifluoroacetic acid (0.8 mL) was added to the protected ligand (0.1 mmol) in dichloromethane (1 mL) and allowed to stir at ambient temperature for 1 h. The reaction mixture was diluted with dichloromethane (5 mL) and excess trifluoroacetic acid was neutralized with saturated NaHCO_3_, the organic layer was separated, dried over anhydrous Na_2_SO_4_, evaporated and concentrated in *vacuo*, and purified as described to isolate the ligand.

### *G-COCH_2_-5-pyridyl-2-NHNHCH_2_CO_2_H (13)*

Following general procedure A, the residue was purified by silica gel column chromatography using MeOH/CH_2_Cl_2_ (10∶ 90) to isolate the product **13** (0.050 g, 96%) as a colorless solid. ^1^H NMR (300 MHz, CD_3_OD) δ 7.82–7.65(m, 4H, Py and Ar), 7.10(s, 1H, Ar), 7.05(s, 1H, Ar), 7.02(d, *J* = 8.8 Hz, 1H, Ar), 6.75(d, *J* = 8.8 Hz, 1H, Ar), 5.99–5.93(m, 3H, OCH_2_O and H-1), 5.65–5.62(m, 1H, H-2), 4.93(d, *J* = 2.9 Hz, 1H, H-4), 4.26(s, 2H, COCH_2_Py), 4.06(d, *J* = 7.2 Hz, 1H, H-9b), 3.67(s, 2H, NCH_2_CO), 3.18–3.10(m, 1H, H-3), 2.49–2.44(m, 1H, H-3), 1.75–1.67(m, 1H, H-3a). ^13^C NMR (75 MHz, CD_3_OD) δ 196.4(CO), 174.0(CO), 163.2(Py), 154.6(Py), 153.2(Py), 149.0(Py), 147.4(Py), 135.3(C-1), 135.1(Ar), 134.9(Ar), 131.5(Ar), 131.1(C-1), 128.7(Ar), 127.4(Ar), 125.90(Ar), 123.3(Ar), 116.3(Ar), 113.7(Ar), 112.0(Ar), 109.2(Ar), 103.3(OCH_2_O), 57.0(C-4), 54.7(NCH_2_CO), 52.4(COCH_2_Py), 46.4(C-9b), 43.5(C-3), 32.4(C-3a); IR (KBr): 3436(NH, OH), 1683(CO), 1622(C = C) cm^-1^; HPLC-MS: Elution with 33% CH_3_CN in H_2_O containing 0.01% formic acid, exhibited single peak at R_t_ = 16.83 min. ESI-MS *m/z* (ES+) calcd [M+H] ^+^ for C_28_H_25_BrN_4_O_5_ 577.20, found 577.19.

### *G-COCH_2_-5-pyridyl-2-CH_2_NHCH_2_CO_2_H* (16)

Following general procedure A, the residue was purified by silica gel column chromatography using MeOH/CH_2_Cl_2_ (20∶ 80) to isolate the product **16** (0.056 g, 70%) as a colorless solid. ^1^H NMR (300 MHz, CDCl_3_) δ 8.34(s, 1H, Py), 7.53(d, *J* = 8.3 Hz, 1H, Py), 7.47(d, *J* = 8.36 Hz, 1H, Py), 7.28(s, 1H, Ar), 7.04(s, 1H, Ar), 6.98(s, 1H, Ar), 6.59(d, *J* = 8.6 Hz, 1H, Ar), 5.96(d, *J* = 1.0 Hz, 1H, OCH_2_O), 5.94(d, *J* = 1.0 Hz, 1H, OCH_2_O), 5.87–5.84(m, 1H, H-1), 5.63–5.57(m, 1H, H-2), 4.89(d, *J* = 2.9 Hz, 1H, H-4), 4.38–4.01(m, 5H, CH_2_, COCH_2_Py, and H-9b), 3.51(s, 2H, NCH_2_CO), 3.17–3.07(m, 1H, H-3), 2.45–2.34(m, 1H, H-3), 1.77–1.69(m, 1H, H-3a); IR (KBr): 3437(NH, OH), 1684(CO), 1597(C = C) cm^−1^; HPLC-MS: Elution with 33% CH_3_CN in H_2_O containing 0.01% formic acid, exhibited single peak at R_t_ = 17.28 min. ESI-MS *m/z* (ES+) calcd [M+H]^+^ for C_29_H_26_BrN_3_O_5_ 576.11, found 576.40.

### *G-CH_2_CH_2_-5-pyridyl-2-NHNHCH_2_CO_2_H* (18)

Following general procedure A, the residue was purified by silica gel column chromatography using MeOH/CH_2_Cl_2_ (10∶ 90) to isolate the product **18** (0.081g, 60%) as a colorless solid consisting of a mixture of syn: anti (3.3∶ 1) determined by NMR; ^1^H NMR (300 MHz, CD_3_OD) δ 7.66(dd, *J* = 9.0, 2.0 Hz, 1H, Py), 7.47(s, 1H, Py), 7.14(s, 1H, Ar), 7.01(s, 1H, Ar), 6.93(d, *J* = 9.0 Hz, 1H, Py), 6.71(s, 1H, Ar), 6.68(d, *J* = 2.0 Hz, 1H, Ar), 6.59(d, *J* = 8.0 Hz, 1H, Ar), 5.97(d, *J* = 1.5 Hz, 1H, OCH_2_O), 5.95(d, *J* = 1.5 Hz, 1H, OCH_2_O), 5.76–5.71(m, 1H, H-1), 5.66–5.55(m, 1H, H-2), 4.73(d, *J* = 3.0 Hz, 1H, H-4), 3.94(d, *J* = 9.0 Hz, 1H, H-9b), 3.61(s, 2H, NCH_2_CO), 3.15–3.05(m, 1H, H-3), 2.84–2.71(m, 4H, ArCH_2_Py), 2.54- 2.46(m, 1H, H-3), 1.71–1.63(m, 1H, H-3a); ^13^C NMR (75 MHz, CD_3_OD) δ 154.2(CO), 148.9(Py), 148.7(Ar), 146.3(Py), 145.5(Py), 136.0(Py), 135.4(C-1), 133.9(Py), 130.7(Ar), 130.1(C-2), 128.6(Ar), 127.4(Ar), 127.0(Ar), 123.9(Ar), 120.0(Ar), 117.4(Py), 116.1(Ar), 113.6(Ar), 113.5(Ar), 112.3(Ar), 112.2(Ar), 109.3(Ar), 103.2(OCH_2_O), 57.9(C-4), 52.8(NCH_2_CO), 47.3(C-9b), 43.5(C-3), 36.8(CH_2_), 34.4(CH_2_), 32.3(C-3a); IR (KBr): 3453(NH and OH), 1682(CO) cm^−1^. HPLC-MS: Elution with 3–93% CH_3_CN (gradient 4.5% min-1) in H_2_O containing 0.01% formic acid, exhibited two peak at Rt = 7.55 and 7.15 min. ESI-MS m/z (ES-) calcd [M−H] - for C_28_H_27_BrN_4_O_4_ 561.12, found 561.33.

### *G-5-pyridyl-2-NHNHCH_2_CO_2_H* (20)

Following general procedure A, the residue was purified by silica gel column chromatography using MeOH/CH_2_Cl_2_(15∶ 85) to isolate the product **20** (0.048 g, 90%) as a colorless solid consisting of a mixture of syn: anti (3∶ 1) determined by NMR; ^1^H NMR (300 MHz, CD_3_OD) δ 8.13(dd, *J* = 9.4, 2.0 Hz, 1H, Py), 7.98(d, *J* = 2.0 Hz, 1H, Py), 7.25(d, *J* = 2.0 Hz, 1H, Py), 7.18–7.14(m, 2H, Ar), 7.08–7.04(m, 2H, Ar), 6.80(d, *J* = 8.5 Hz, 1H, Ar), 6.00(d, *J* = 1.1 Hz, 1H, OCH_2_O), 5.98(d, *J* = 1.1 Hz, 1H, OCH_2_O), 5.95–5.92(m, 1H, H-1), 5.65–5.62(m, 1H, H-2), 4.83–4.82(m, 1H, H-4), 4.08(d, *J* = 8.8 Hz, 1H, H-9b), 3.62(s, 2H, NCH_2_CO), 3.21–3.13(m, 1H, H-3), 2.57–2.48(m, 1H, H-3), 1.75–1.67(m, 1H, H-3a); IR (KBr): 3435(NH and OH), 1678(CO)cm^−1^; HPLC-MS: Elution with 3–93% CH_3_CN (gradient 4.5% min^−1^) in H_2_O containing 0.01% formic acid, exhibited a single peak R_t_ = 6.22 min. ESI-MS *m/z* (ES+) calcd for C_26_H_23_BrN_4_O_4_ (M+H)^+^ 535.09; found 535.07.

### *G-triazole-5-pyridyl-2-NHNHCH_2_CO_2_H* (23)

Following general procedure A, the residue was purified by silica gel column chromatography using MeOH/CH_2_Cl_2_(15∶ 85) to isolate the product **23** (0.058 g, 96%) as a colorless solid. ^1^H NMR (300 MHz, CD_3_OD) δ 8.36(s, 1H, Tri), 8.31–8.21(m, 2H, Ar), 7.40(d, *J* = 2.2 Hz, 1H, Py), 7.29(dd, *J* = 8.6, 2.5 Hz, 1H, Py), 7.18(d, *J* = 7.2 Hz, 1H, Py), 7.10(s, 1H, Ar), 7.0(s, 1H, Ar), 6.73(d, *J* = 8.5 Hz, 1H, Ar), 5.97(d, *J* = 1.1 Hz, 1H, OCH_2_O), 5.96(d, *J* = 1.1 Hz, 1H, OCH_2_O), 5.90–5.86(m, 1H, H-1), 5.70–5.65(m, 1H, H-2), 4.89(d, *J* = 3.0 Hz, 1H, H-4), 4.12(d, *J* = 8.5 Hz, 1H, H-9b), 3.52(s, 2H, NCH_2_CO), 3.24–3.08(m, 1H, H-3), 2.57–2.50(m, 1H, H-3), 1.83–1.76(m, 1H, H-3a); IR (neat): 1696 (CO) cm^−1^HPLC-MS: Eluting with 10–90% CH_3_CN (gradient 2.66% min^−1^) in H_2_O exhibited single peak at R_t_ = 23.17 min. ESI-MS *m/z* (ES-) calcd [M−H]^−^ for C_28_H_24_BrN_7_O_4_ 600.11, found 600.09.

### *G-triazole-CH_2_NHCH_2_CO_2_H* (25)

Following general procedure A, the residue was purified by addition of water, producing a solid that was filtered and purified by silica gel column chromatography using MeOH/CH_2_Cl_2_ (20∶ 70) to isolate the product **25** (0.039 g, 75%) as a white solid. ^1^H NMR (300 MHz, CD_3_OD) δ 8.41(s, 1H, Py), 7.37(d, *J* = 2.5 Hz, 1H, Py), 7.29(dd, *J* = 8.6, 2.5 Hz, 1H, Py), 7.14(s, 1H, Ar), 7.01(s, 1H, Ar), 6.83(d, *J* = 8.6 Hz, 1H, Ar), 5.98(d, *J* = 1.1 Hz, 1H, OCH_2_O), 5.97(d, *J* = 1.1 Hz, 1H, OCH_2_O), 5.90–5.85(m, 1H, H-1), 5.67–5.63(m, 1H, H-2), 4.86(d, *J* = 3.0 Hz, 1H, H-4), 4.43(s, 2H, TriCH_2_N), 4.08(d, *J* = 8.6 Hz, 1H, H-9b), 3.62(s, 2H, NCH_2_CO), 3.23–3.12(m, 1H, H-3), 2.57–2.49(m, 1H, H-3), 1.79–1.70(m, 1H, H-3a); IR (KBr): 3430(OH), 1683(CO) cm^−1^; HPLC-MS: Eluting with 10–90% CH_3_CN (gradient 3% min^−1^) in H_2_O, exhibited single peak at R_t_ = 18.85 min. ESI-MS *m/z* (ES+) calcd [M+H]^+^ for C_24_H_22_BrN_5_O_4_ 524.09, found 524.32.

### General procedure B; complexation with Re(CO)_3_^+^

A mixture of ReBr(CO)_3_(H_2_O)_3_(1.1 eq) and NaHCO_3_(1.0 eq) in water was added to the ligand in EtOH and allowed to stir at rt for 3–6 h. Volatiles were removed, water was added, the resulting precipitate was filtered and washed with water. The residue was purified as described to isolate the rhenium complex.

### *G-COCH_2_-5-pyridyl-2-NHNHCH_2_CO_2_Re(CO)_3_* (5-Re)

Following the general procedure B using EtOH/H_2_O (1.3∶ 1), crude residue was purified by silica gel column chromatography using MeOH/CH_2_Cl_2_ (05∶ 95) to isolate the complex **5** (0.063 g, 75%) as a white solid. ^1^H NMR (300 MHz, DMSO-*d*_6_) δ 10.01(s, 1H, NH), 9.50 (s, 1H, NH), 8.14(s, 1H, Py), 7.74(s, 1H, Py), 7.62(d, *J* = 8.5 Hz, 2H, Py), 7.25(s, 1H, Ar), 7.11(s, 1H, Ar), 6.84–6.76(m, 2H, Ar), 6.54(s, 1H, NH), 6.10(s, 1H, OCH_2_O), 6.07(s, 1H, OCH_2_O), 6.02–5.97(m, 1H, H-1), 5.64–5.58(m, 1H, H-2), 4.80(d, *J* = 2.6 Hz, 1H, H-4), 4.33–4.16(m, 2H, COCH_2_Py), 4.06(d, *J* = 8.5 Hz, 1H, H-9b), 3.75–3.61(m, 2H, NCH_2_CO), 3.07–2.98(m, 1H, H-3), 2.45–2.35(m, 1H, H-3), 1.71–1.63(m, 1H, H-3a); ^13^C NMR (75 MHz, DMSO-*d*_6_) δ 197.8(CO), 197.2(CO), 196.5(CO), 194.5(CO), 176.6(CO), 158.0(Py), 151.1(Ar), 148.5(Py), 147.2(Py), 147.1(Ar), 142.5(Ar), 134.5(C-1), 133.3(Ar), 130.1(Ar), 130.0(Ar), 129.6(C-2), 127.1(Ar), 125.9(Ar), 123.6(Ar), 123.1(Ar), 115.1(Ar), 112.2(Ar), 112.2(Ar), 108.4 (Ar), 107.0 (Ar), 101.9(OCH_2_O), 58.6(C-4), 55.2(N-CH_2_-CO),44.7(CO-CH_2_-Py), 41.7(C-3), 31.2 (C-3a); IR (KBr): 3429(NH), 2027(CO), 1918(CO), 1893(CO), 1630(CO) cm^−1^; HPLC-MS: Eluting with 63–93% CH_3_CN (gradient 1% min^−1^) in H_2_O containing 0.01% formic acid, exhibited single peak at R_t_ = 16.32 min. ESI-MS *m/z* (ES+) calcd [M+H] ^+^ for C_31_H_24_BrN_4_O_8_Re 847.04, found 847.30. HRMS: calcd [M+H]^+^ for C_31_H_24_BrN_4_O_8_Re 847.0413, found 847.0391.

### *G-COCH_2_-5-pyridyl-2-CH_2_NHCH_2_CO_2_Re(CO)_3_* (6-Re)

Following the general procedure B using EtOH/H_2_O (2∶ 1), the crude residue was purified by silica gel column chromatography using MeOH/CH_2_Cl_2_ (8∶ 92) to isolate the product **6** (0.030 g, 60%) as a white solid ^1^H NMR (300 MHz, DMSO-*d*_6_) δ 8.71–8.69(m, 1H, Py), 7.97–7.94(m, 1H, Py), 7.76(bs, 1H, NH), 7.65(d, *J* = 7.7 Hz, 2H, Ar), 7.25(s, 1H, Ar), 7.22–7.18(m, 1H, Ar), 7.12(s, 1H, Ar), 6.79(d, *J* = 7.9 Hz, 1H, Ar), 6.58(s, 1H, Ar), 6.10(d, *J* = 0.7 Hz, 1H, OCH_2_O), 6.08(d, *J* = 0.7 Hz, 1H, OCH_2_O), 6.04–5.98(m, 1H, H-1), 5.65–5.59(m, 1H, H-2), 4.82(d, *J* = 3.0 Hz, 1H, H-4), 4.60–4.38(m, 4H, COCH_2_Py and NCH_2_CO), 4.08(d, *J* = 8.5 Hz, 1H, H-9b), 3.62–3.53(m, 1H, Py-CH_2_-N), 3.25–3.20(m, 1H, Py-CH_2_-N), 3.09–2.99(m, 1H, H-3), 2.44–2.35 (m, 1H, H-3), 1.71–1.63(m, 1H, H-3a); ^13^C NMR (75 MHz, CDCl_3_) δ 197.6(CO), 197.4(CO), 197.4(CO), 193.8(CO), 179.4(CO), 157.8(Py), 152.5(Py), 151.2(Ar), 147.2(Py), 147.1(Py), 141.7(Py), 134.5(C-1), 133.7(Ar), 133.2(Ar), 130.1(Ar), 129.7(C-2), 127.2(Ar), 125.8(Ar), 123.7(Ar), 122.8(Ar),115.7(Ar),112.4(Ar),112.2(Ar),108.4(Ar),104.4(Ar),102.0(OCH_2_O), 61.7(C-4), 55.2(NCH_2_CO), 53.9(PyCH_2_N), 44.7(COCH_2_Py), 41.7(C-3), 31.2 (C-3a); IR (KBr): 3429(NH), 2023(CO), 1920(CO), 1889(CO), 1635(CO) cm^−1^; HPLC-MS: Eluting with 63–93% CH_3_CN in H_2_O (gradient 1% min^−1^) containing 0.01% formic acid, exhibited single peak at R_t_ = 20.63 min. ESI-MS *m/z* (ES+) calcd [M+H]^+^ for C_32_H_25_BrN_3_O_8_Re 846.04, found 846.31; HRMS: Calcd [M+H]^+^ for C_32_H_25_BrN_3_O_8_Re 846.0461, found 846.0428.

### *G-CH_2_CH_2_-5-pyridyl-2-NHNHCH_2_CO_2_Re(CO)_3_* (7-Re)

Following the general procedure B using EtOH/H_2_O (3∶1) the crude residue was purified by silica gel column chromatography using MeOH/CH_2_Cl_2_ (94∶ 06) to isolate the complex **7** (0.051 g, 62%) as a white solid consisting of a mixture of syn: anti (4.5∶ 1) determined by NMR; ^1^H NMR (300 MHz, DMSO-*d_6_*) δ 9.93(s, 1H, NH), 9.46(s, 1H, NH), 7.96(d, *J* = 5.6, 1.8 Hz, 1H, Py), 7.65(d, *J* = 8.8, 2.0 Hz, 1H, Py), 7.22(s, 1H, Ar), 7.13(s, 1H, Ar), 7.13(s, 1H, Ar), 6.87–6.72(m, 3H, Ar and Py), 6.63–6.59(m, 1H, Ar), 6.08(d, *J* = 0.8 Hz, 1H, OCH_2_O), 6.06(d, *J* = 0.8 Hz, 1H, OCH_2_O), 5.84–5.81(m, 1H, H-1), 5.59–5.56(m, 1H, H-2), 5.40(d, *J* = 9.4 Hz, 1H, H-4), 4.63(d, *J* = 2.9 Hz, 1H, H-9b), 3.97–3.94(m, 1H, NCH_2_CO), 3.67–3.65(m, 1H, NCH_2_CO), 3.01–2.95(m, 1H, H-3), 2.77–2.65(m, 5H, H-3 and CH_2_), 1.67–1.58(m, 1H, H-3a); IR (KBr): 3428(NH), 2025(CO), 1916(CO), 1887(CO), 1631(CO) cm^−1^; HPLC-MS: Eluting with 3–93% CH_3_CN (gradient 4.5% min^−1^) in H_2_O containing 0.01% formic acid, exhibited single peak at R_t_ = 17.68 min. ESI-MS *m/z* (ES+) calcd [M+H] ^+^ for C_31_H_26_BrN_4_O_7_Re 833.06 found 833.19. HRMS: Calcd [M+H]^+^ for C_31_H_26_BrN_4_O_7_Re 833.0621, found 833.0602.

### *G-5-pyridyl-2-NHNHCH_2_CO_2_Re(CO)_3_* (8-Re)

Following the general procedure B using EtOH/H_2_O (2∶ 1). The crude complex was purified by column chromatography using MeOH/CH_2_Cl_2_ (8∶ 92) to isolate the product **8** (0.069 g, 87%) as a white solid. ^1^H NMR (400 MHz, DMSO-*d*_6_) δ 10.11(s, 1H, NH), 9.57(s, 1H, NH), 8.25(d, *J* = 1.6 Hz, 1H, Py), 7.98(d, *J* = 7.2 Hz, 1H, Py), 7.27–7.15(m, 4H, Ar), 6.91(d, *J* = 8.2 Hz, 1H, Ar), 6.81(d, *J* = 7.2 Hz, 1H, Ar), 6.10(d, *J* = 1.1 Hz, 1H, OCH_2_O), 6.08(d, *J* = 1.1 Hz, 1H, OCH_2_O), 5.98–5.94(m, 1H, H-1), 5.86(bs, 1H, NH), 5.63–5.58(m, 1H, H-2), 4.70(d, *J* = 2.8 Hz, 1H, H-4), 4.09(d, *J* = 9.0 Hz, 1H, H-9b), 3.69(m, 2H, N-CH_2_-CO), 3.06–3.03(m, 1H,H-3), 2.48–2.42(m, 1H, H-3), 1.72–1.63(m, 1H, H-3a); IR (KBr): 3252(NH), 2026(CO), 1916(CO), 1898(CO), 1667(CO) cm^−1^; HPLC-MS: Eluting with 3–93% CH_3_CN (gradient 4.5% min^−1^) in H_2_O containing 0.01% formic acid, exhibited single peak at R_t_ = 15.37 min. ESI-MS *m/z* (ES+) calcd [M−H]^+^ for C_29_H_22_BrN_4_O_7_Re 803.02 found 803.07; HRMS: Calcd [M+H] ^+^ for C_29_H_22_BrN_4_O_7_Re 805.0308, found 833.0309.

### *G-triazole-5-pyridyl-2-NHNHCH_2_CO_2_Re(CO)_3_* (9-Re)

Following the general procedure B using EtOH/H_2_O (2∶ 1) the crude residue was purified by silica gel (SiO_2_) flash column chromatography using MeOH/CH_2_Cl_2_ (05∶ 95) to isolate the complex **9** (0.060 g, 69%) as a yellow solid ^1^H NMR (300 MHz, CD_3_OD) δ 8.79(d, *J* = 2.0 Hz, 1H, Py), 8.69(s, 1H, Tri), 8.17(dd, *J* = 8.9, 2.0 Hz, 1H, Py), 7.48(d, *J* = 2.0 Hz, 1H, Py), 7.38(dd, *J* = 8.3, 2.0 Hz, 1H, Ar), 7.15(s, 1H, Ar), 7.03(s, 1H, Ar), 6.92(d, *J* = 8.8 Hz, 1H, Ar), 6.84(d, *J* = 8.8 Hz, 1H, Ar), 5.99(d, *J* = 1.1 Hz, 1H, OCH_2_O), 5.98(d, *J* = 1.1 Hz, 1H, OCH_2_O), 5.96–5.92(m, 1H, H-1), 5.70–5.65(m, 1H, H-2), 4.88(d, *J* = 3.0 Hz, 1H, H-4), 4.12(d, *J* = 9.0 Hz, 1H, H-9b), 3.89(s, 2H, NCH_2_CO), 3.21–3.14(m, 1H, H-3), 2.58–2.50(m, 1H, H-3), 1.80–1.71(m, 1H, H-3a); HPLC-MS: Eluting with 40–90% CH_3_CN (gradient 1.5% min^−1^) in H_2_O exhibited single peak at R_t_ = 8.22 min. ESI-MS *m/z* (ES+) calcd [M+H]^+^ for C_31_H_23_BrN_7_O_7_Re 872.04, found 872.05.

### *G-triazole-CH_2_NHCH_2_CO_2_Re(CO)_3_* (10-Re)

A mixture of ReBr(CO)_3_(H_2_O)_3_ (0.044 g, 0.11 mmol) and NaHCO_3_ (0.008 g, 0.10 mmol) in water (5 mL) was added to the triazole ligand **25** (0.052 g, 0.10 mmol) in EtOH (10 mL) heated at 65°C for 1 h. The reaction mixture was cooled and solvents were evaporated in vacuo, water (10 mL) was added, and the solids were separated by filtration and washed with water (30 mL) to isolate the complex **10** (0.073 g, 92%). ^1^H NMR (300 MHz, DMSO-*d*_6_) δ 8.85(d, *J* = 4.0 Hz, 1H, tri), 7.56(d, *J* = 2.5 Hz, 1H, Ar), 7.45–7.38(m, 2H, Ar), 7.26(s, 1H, Ar), 7.15(s, 1H, Ar), 6.89(d, *J* = 8.4 Hz, 1H, Ar), 6.25(s, 1H, NH), 6.10(s, 1H, OCH_2_O), 6.08(s, 1H, OCH_2_O), 6.00–5.97(m, 1H, H-1), 5.63–5.62(m, 1H, H-1), 4.76(d, *J* = 3.8 Hz, 1H, H-4), 4.35–4.25(m, 2H, CH_2_CO), 4.12(d, *J* = 8.4 Hz, 1H, H-9b), 3.63–3.57(m, 1H, NCH_2_Tri), 3.37–3.35(m, 2H, N-CH_2_Tri and NH), 3.10–3.01(m, 1H, H-3), 2.44–2.35(m, 1H, H-3), 1.72–1.66(m, 1H, H-3a); ^13^C NMR (75 MHz, DMSO-*d*_6_) δ 197.4(CO), 196.9(CO), 196.6(CO), 179.1(CO), 148.9(Tri), 147.8(Tri), 147.19(Ar), 147.14(Ar), 134.2(Ar), 133.5(Ar), 130.0(Ar), 126.3(Ar), 125.7(Ar), 121.5(Ar), 121.1(Ar), 119.1(Ar), 116.4(Ar), 112.4(Ar), 112.2(C-1), 108.4(C-2), 101.9(OCH_2_O), 55.6(C-4), 55.0(N-CH_2_-CO), 51.8(TriCH_2_N), 45.2(C-9b), 41.6(C-3), 31.2(C-3a). IR (KBr): 2025(CO), 1990(CO), 1675(CO) cm^−1^; HPLC-MS: Eluting with 10–90% CH_3_CN (gradient 3% min^−1^) in H_2_O, exhibited single peak at R_t_ = 25.15 min. ESI-MS *m/z* (ES+) calcd [M+H]^+^ for C_27_H_21_BrN_5_O_7_Re 794.04, found 794.26; HRMS: Calcd [M+H]^+^ for C_27_H_21_BrN_5_O_7_Re 794.0255, found 794.0258.

### *Cell culture*

ER α/β-negative and GPER-expressing human breast carcinoma SKBr3 cells and ER α/β and GPER-negative monkey kidney COS7 cells were cultured in DMEM tissue media (COS7) or RPMI-1640 tissue media (SKBr3), with fetal bovine serum (10%), 2 mM L-glutamine, 100 units/mL penicillin and 100 µg/mL streptomycin. Cells were grown as a monolayer at 37°C, in a humidified atmosphere of 5% CO_2_ and 95% air. Lipofectamine 2000 was used according to manufacturer's directions for all transfections except PH-RFP was transfected at ¼ the recommended amount to achieve reasonable expression levels.

### *Intracellular calcium mobilization*

SKBr3 cells (1×10^7^/mL) were incubated in HBSS containing 3 µM Indo1-AM (Invitrogen) and 0.05% pluronic acid F-127 for 1 h at RT. Cells were then washed twice with HBSS, incubated at RT for 20 min, washed again with HBSS, resuspended in HBSS at a density of 10^8^cells/mL and kept on ice until assay, performed at a density of 2×10^6^cells/mL. Ca^++^ mobilization was determined ratiometrically using λ_ex_ 340 nm and λ_em_ 400/490 nm at 37°C in a spectrofluorometer (QM-2000–2, Photon Technology International) equipped with a magnetic stirrer.

### *PI3K activation*

The PIP3 binding domain of Akt fused to mRFP1 (PH-RFP) was used to localize cellular PIP3. COS7 cells co-transfected with GPER-GFP or ERα-GFP and PH-RFP were plated on coverslips and serum starved for 24 h followed by stimulation with ligands as indicated. The cells were fixed with 2% PFA in PBS, washed, mounted in Vectashield containing DAPI (Vector Labs) and analyzed by confocal microscopy using a Zeiss LSM510 confocal fluorescent microscope.

### *Receptor binding*

Binding assays for ERα and ERβ were performed as previously described [30]. Briefly, COS7 cells were transiently transfected with ERα-GFP or ERβ-GFP. Following serum starvation for 24 h, cells (∼5×10^4^) were incubated with Re-labeled derivatives for 10 min in a final volume of 10 µL prior to addition of 10 µL of 20 nM E2-Alexa633 in saponin-based permeabilization buffer. Following 5 min at RT, cells were washed once with 1 mL PBS/2%BSA, resuspended in 200 µL and analyzed on a FACS Calibur flow cytometer (BD Biosciences).

### ^99m^Tc-radiolabeling

The carbonyl complex [^99m^Tc(CO)_3_(H_2_O)_3_]+ was prepared by adding 3.7 GBq of freshly eluated Na-^99m^TcO_4_ to the Isolink® kit (Tyco healthcare, Mallinckrodt, St. Louis, MO, USA) as previously described [20], The alkaline [^99m^Tc(CO)_3_(H_2_O)_3_]^+^ mixture was then neutralized to pH 7 with acetic acid. The ligands were dissolved in ethanol and 10 µg of each of the derivatives was added to the prepared [^99m^Tc(CO)_3_(H_2_O)_3_]^+^ mixture. The reaction mixture was stirred for 2 h at room temperature. The inorganic impurities from Isolink® kit, aqua ions of ^99m^Tc (if any) and excess ligand were separated using solid phase extraction (SPE) technique. SPE was performed using C-18 SepPak Plus cartridges (Waters, Milford, MA USA). The impurities and excess of ligand were eluted with 4 fractions of 0.5 mL of the weak solvent (50% ethanol in water). Elution of the final ^99m^Tc-labeled complexes was performed with 4 fractions of 0.5 mL strong solvent (100% ethanol). HPLC was performed to assess radiochemical purity and specific activity. Ten µL of the final product was added to 200 µL of HPLC grade ethanol (JT Baker, Phillipsburg, NJ, USA). To asses radiochemical purity and specific activity, 10 µL of the diluted quality control sample was injected on a reverse-phase C-18 column (JT Baker, Phillipsburg, NJ, USA) using HPLC grade ethanol and HPLC grade water as previously described [22]. Stability, transchelation and partition coefficient studies were performed as previously described [22].

## Supporting Information

Text S1 **Additional experimental details for synthesis of ligands 13, 16, 18, 20, 23, 25.**
(DOC)

Figure S1 **Synthetic scheme for preparation of 5-ethynyl derivative 12.**
(TIF)

Figure S2 **Synthetic scheme for preparation of G-CC-5-pyridyl-2-N(** ***^t^*** **Boc)N(** ***^t^*** **Boc)CH_2_CO_2_** ***^t^*** **Bu.**
(TIF)

Figure S3 **Synthetic scheme for preparation of picoline amine derivative 15.**
(TIF)

Figure S4 **Synthetic scheme for preparation of** ***tert*** **-butyl-2-(** ***tert*** **-butoxycarbonyl((5-ethynylpyridine-2-yl)methyl)amino)ethanoate.**
(TIF)

Figure S5 **Synthetic scheme for preparation of G-CC-5-pyridyl-2-CH_2_-N(** ***^t^*** **Boc)CH_2_CO_2_** ***^t^*** **Bu.**
(TIF)

Figure S6 **Synthetic scheme for preparation of aniline derivative 17.**
(TIF)

Figure S7 **Synthetic scheme for preparation of G-CH_2_CH_2_-5-pyridyl-2-N(** ***^t^*** **Boc)-N(** ***^t^*** **Boc)CH_2_CO_2_** ***^t^*** **Bu.**
(TIF)

Figure S8 **Synthetic scheme for preparation of aniline derivative 19.**
(TIF)

Figure S9 **Synthetic scheme for preparation of G-5-pyridyl-2-N(** ***^t^*** **Boc) N(** ***^t^*** **Boc)CH_2_CO_2_** ***^t^*** **Bu.**
(TIF)

Figure S10 **Synthetic scheme for preparation of azide product 22.**
(TIF)

Figure S11 **Synthetic scheme for preparation of G-triazole-5-pyridyl-2-N(** ***^t^*** **Boc) N(** ***^t^*** **Boc)CH_2_CO_2_** ***^t^*** **Bu.**
(TIF)

Figure S12 **Synthetic scheme for preparation of G-triazole-CH_2_N(^t^Boc)CH_2_CO_2_** ***^t^*** **Bu.**
(TIF)
