# Supplementary material for: Synthesis and Characterization of Tricarbonyl-Re/Tc(I) Chelate Probes Targeting the G Protein-Coupled Estrogen Receptor GPER/GPR30
Source: PLoS One. 2012 Oct 15;7(10):e46861. doi: 10.1371/journal.pone.0046861 (PMC3471960; doi:10.1371/journal.pone.0046861)
Supplement: Text S1 — Additional experimental details for synthesis of ligands 13, 16, 18, 20, 23, 25. (DOC) [file pone.0046861.s001.doc]

Supporting Information

for

Synthesis and characterization of tricarbonyl-Re/Tc(I) chelate probes targeting the G protein-coupled estrogen receptor GPER/GPR30

Ritwik Burai,**^1^** Chinnasamy Ramesh,**^1^** Tapan K. Nayak,**^2^** Megan K. Dennis,**^2^** Bj K. Bryant,**^1^** Eric R. Prossnitz,**^2,3^** and Jeffrey B. Arterburn**^1,3^**^*^

**1** Department of Chemistry and Biochemistry, New Mexico State University, Las Cruces, New Mexico, United States of America, **2** Department of Cell Biology and Physiology, University of New Mexico Health Science Center, Albuquerque, New Mexico, United States of America, **3** University of New Mexico Cancer Center, University of New Mexico Health Science Center, Albuquerque, New Mexico, United States of America

**Contents: Additional experimental details for synthesis of ligands 13, 16, 18, 20, 23, 25.**

1. Synthesis of chemical intermediates for ligand **13**……………………………………S2-S3

2. Synthesis of chemical intermediates for ligand **16**……………………………………S4-S6

3. Synthesis of chemical intermediates for ligand **18**……………………………………S7-S8

4. Synthesis of chemical intermediates for ligand **20**……………………………………S9-S10

5. Synthesis of chemical intermediates for ligand **23, 25**……………………….……….S11-S13

6. References cited……………………………………………………….........................S13

1. Synthesis of chemical intermediates for ligand **13** (Refer to Figures S1 and S2).

Bromide **11** (1.557 g, 3.10 mmol), PdCl_2_ (PPh_3_)_2_ (0.217 g, 0.31 mmol, 10 mol %), CuI (0.047 g, 0.247 mmol, 8 mol %) weighed into round bottom flask, flushed with argon. N-Methylpyrrolidone (NMP) (5 mL) was added followed by the addition of ethynyltrimethylsilane (3.56 mL, 24.8 mmol) and Hunig’s base (1.4 mL) and stirred at 80 ^0^C under argon for 2 h. The reaction mixture was cooled; water (30 mL) was added and extracted with EtOAc (50 mL). The residue was purified by silica gel column chromatography using EtOAc/hexanes (8: 92) to isolate the di-*tert*-butyl 1-(2-*tert*-butoxy-2-oxoethyl)-2-(5-((trimethylsilyl)ethynyl)pyridin-2-yl)hydrazine-1,2-dicarboxylate (1.514 g, 94%) as a brownish oil. ^1^H NMR (300 MHz, CDCl_3_, mixture of carbamate rotamers observed) δ 8.36-8.35 (m, 1H, Py), 7.66-7.62 (m, 1H, Py), 7.56-7.51 (m, 1H, Py), 4.20-4.13 (m, 1H, CH_2_), 4.06-3.95 (m, 1H, CH_2_), 1.46-1.32 (m, 27H, CH_3_), 0.18-0.17 (m, 9H, SiCH_3_); ^13^C NMR (75 MHz, CDCl_3_) δ 166.7(CH_2_CO), 166.6(CH_2_CO), 154.2(CO), 153.6(CO), 152.1(CO), 152.0(CO), 151.9(Py), 151.6(Py), 150.2(Py), 150.1(Py), 139.9(Py), 139.6(Py), 116.8(Py), 116.5(Py), 116.1(Py), 115.9(Py), 101.2(Alkyne), 101.1(Alkyne), 96.8(Alkyne), 96.7(O-C), 82.3(O-C), 81.6(O-C), 81.0(O-C), 80.5(O-C), 76.3(O-C), 54.4(CH_2_), 52.4(CH_2_), 27.6(CH_3_), 27.6(CH_3_), 27.5(CH_3_),-0.55 (SiCH_3_); IR (KBr): 2966(C-H), 2158 (alkyne), 1690 (C=O), 1719 (C=O),cm^-1^. Silver triflate (0.140 g, 0.54 mmol, 20 mol%) was added to the TMS-ethyne (1.406 g, 2.71 mmol) in 12 mL of CH_2_Cl_2_/MeOH/H_2_O (7: 4: 1) and allowed to stir at room temperature for 20 h. The reaction mixture was diluted with saturated NaHCO_3_ (30 mL), the product was extracted using dichloromethane (3 x 20 mL) and evaporated under reduced pressure. The residue was purified by silica gel column chromatography using EtOAc/hexanes (10: 90) to isolate the product **12** (0.970 g, 80%) as colorless oil. ^1^H NMR (300 MHz, CDCl_3_, mixture of carbamate rotamers observed) δ 8.36-8.34 (m, 1H, Py), 7.66-7.62 (m, 1H, Py), 7.55-7.50 (m, 1H, Py), 4.17-4.12 (m, 1H, CH_2_), 4.00-3.91 (m, 1H, CH_2_), 3.14 (m, 1H, Alkyne), 1.43-1.27 (m, 27H, CH_3_); ^13^C NMR (50 MHz, CDCl_3_) δ 166.8(CH_2_CO), 166.7(CH_2_CO), 154.3(CO), 153.7(CO), 152.4(CO), 152.2(CO), 152.1(Py), 152.0(Py), 150.5(Py), 150.4(Py), 140.2(Py), 140.0(Py), 117.0(Py), 116.2(Py), 115.69(Py), 115.3(Py), 82.5(alkyne), 81.8(alkyne), 81.2(O-C), 80.7(O-C), 80.0(O-C), 79.9(O-C), 79.9(O-C), 77.1(O-C), 54.5(CH_2_), 52.6(CH_2_), 27.7(CH_3_), 27.7(CH_3_), 27.6(CH_3_); IR (KBr):, 1719(CO) cm^-1^.

Iodide **1** (0.150 g, 0.302 mmol), alkyne **12** (0.160 g, 0.357 mmol), PdCl_2_(PPh_3_)_2_  (0.022 g, 0.03 mmol, 10 mol %) and copper (I) iodide (0.006 g, 0.03 mmol, 10 mol %) weighed into a 25 mL round bottomed flask. NMP (2 mL) and dry Et_3_N (0.5 mL) was added, allowed to stir at room temperature under argon for 6 h. The reaction mixture diluted with water (35 mL) and the product was extracted with ethyl acetate (60 mL), evaporated under reduced pressure. The residue was purified by silica gel column chromatography using EtOAc/hexanes (8: 92) to isolate the product G-CC-5-pyridyl-2- N(*^t^*Boc)N(*^t^*Boc)CH_2_CO_2_*^t^*Bu (0.195 g, 79%) as a colorless solid; ^1^H NMR (300 MHz, CDCl_3_) δ 8.49-8.45 (m, 1H, Py), 7.78-7.72 (m, 1H, Py), 7.61-7.56 (m, 1H, Py), 7.26-7.23 (m, 1H, Py), 7.16 (dd, *J* = 8.2, 2.0 Hz, 1H, Ar), 7.13 (s, 1H, Ar), 7.03 (s, 1H, Ar), 6.59-6.56 (m, 1H, Ar), 6.00 (d, *J* = 1.5 Hz, 1H, OCH_2_O), 5.99 (d, *J* = 1.5 Hz, 1H, OCH_2_O), 5.91-5.88 (m, 1H, H-1), 5.69-5.67 (m, 1H, H-2), 4.93 (d, *J* = 3.0 Hz, 1H, H-4), 4.33 (s, 1H, NCH_2_CO), 4.27 (s, 1H, NCH_2_CO), 4.12-4.01 (m, 1H, H-9b), 3.47 (bs, 1H, NH), 3.22-3.16 (m, 1H, H-3), 2.59-2.50 (m, 1H, H-3), 1.86-1.77 (m, 1H, H-3a), 1.54-1.39 (m, 27H, CH_3_); ^13^C NMR (50 MHz, CDCl_3_) δ 167.2(CH_2_CO), 167.1(CH_2_CO), 154.6(CO), 154.5(CO), 152.3(CO), 151.7(CO), 151.3(Py), 149.8(Py), 149.8(Py), 147.4(Py), 147.2(Ar), 145.9(Ar), 145.8(Ar), 139.6(Py), 139.3(Py), 133.8(C-1), 133.6(Ar), 132.4(Ar), 130.3(Ar), 129.7(C-2), 125.8(Ar), 117.9(Py), 117.59(Py), 117.5(Py), 116.6(Py), 115.8(Py), 112.8(Ar), 112.7(Ar), 112.5(Ar), 112.5(Ar), 107.7(Ar), 101.7(OCH_2_O), 92.9(alkyne), 83.9(alkyne), 83.8(O-C), 82.6(O-C), 81.9(O-C), 81.4(O-C), 81.0(O-C), 77.2(O-C), 56.2(C-4) 54.8(NCH_2_CO), 53.0(NCH_2_CO), 45.4(C-9b), 41.9(C-3), 31.2(C-3a), 28.0(CH_3_), 27.9(CH_3_), 27.8(CH_3_), 27.8(CH_3_); IR (KBr):, 2106(alkyne), 1724 (CO) cm^-1^.

2. Synthesis of chemical intermediates for ligand **16** (Refer to Figures S3, S4 and S5).

To a solution of 5-bromo-2-methylpyridine **14** (1.72 g, 10 mmol) and N-bromosuccinimide (1.78 g, 10 mmol) in CCl_4_ (20 mL), AIBN (0.328 g, 2 mmol, 20 mol %) was added and heated at 65 ^0^C for 20 h. The reaction mixture was cooled, filtered and washed with CCl_4_ (10 mL). The volatiles were evaporated and the residue was purified by silica gel column chromatography using EtOAc/hexanes (5: 95) to isolate 5-bromo-2-(bromomethyl)pyridine (1.1 g, 43%) as colorless solid. ^1^H NMR (300 MHz, CDCl_3_) δ 8.62 (d, *J* = 2.0 Hz, 1H, Py), 7.81 (dd, *J* = 8.5, 2.0 Hz, 1H, Py), 7.34 (d, *J* = 8.5 Hz, 1H, Py), 4.5 (s, 2H, CH_2_); ^13^C NMR (75 MHz, CDCl_3_) δ 155.2(Py), 150.5(Py), 139.5(Py), 124.6(Py), 119.9(Py), 32.6(CH_2_); IR (KBr):1702(CO), 1467, 1367 cm^-1^. The *tert*-butoxycarbonylamino-acetic acid *tert*-butyl ester (0.92 g, 3.98 mmol) was dissolved in dry DMF (3 mL) and cooled to 0 °C. Sodium hydride (0.192 g, 8 mmol) was added, followed by a solution of 5-bromo-2-(bromomethyl)pyridine (1.0 g, 3.98 mmol) in DMF (1 mL). The reaction mixture was allowed to stir at room temperature for 2 h under argon. The reaction mixture was quenched with ice-cold water (1 mL), diluted with de-ionized water (25 mL), the product was extracted using ethyl acetate (3x 20 mL), dried over anhydrous sodium sulphate, concentrated and dried. The crude residue was purified by silica gel column chromatography using EtOAc/hexanes (08: 92) to isolate the product **15** (1.32 g, 83%) as a colorless oil. ^1^H NMR (300 MHz, CDCl_3_) δ 8.57-8.54 (m, 1H, Py), 7.82-7.76 (m, 1H, Py), 7.35-7.25 (m, 1H, Py), 4.57 (s, 1H, PyCH_2_), 4.52 (s, 1H, PyCH_2_), 3.99 (s, 1H, NCH_2_), 3.87 (s, 1H, NCH_2_), 1.46-1.41(m, 18H, CH_3_); ^13^C NMR (75 MHz, CDCl_3_) δ 168.5(CO), 168.3(CO), 156.5(Py), 156.2(Py), 155.1(Py), 154.9(Py), 149.7(Py), 149.6(Py), 138.9(Py), 138.7(Py), 123.5(Py), 122.4(Py), 118.9(Py), 118.6(Py), 81.0(O-C), 80.9(O-C), 80.1(O-C), 80.0(O-C), 53.0(NCH_2_), 52.6(NCH_2_), 49.7(PyCH_2_), 49.5(PyCH_2_), 27.8(CH_3_), 27.6(CH_3_); IR (Neat): 1737(CO), 1707(CO) cm^-1^.

Bromide **15** (1.00 g, 2.49 mmol), PdCl_2_(PPh_3_)_2_  (0.174 g, 0.249 mmol, 10 mol %), CuI (0.378 g, 0.199 mmol, 8 mol%) were combined in a 25 mL round bottomed flask. NMP (5 mL) was added, followed by the addition of ethynyltrimethylsilane (2.86 mL, 19.92 mmol), Hunig’s base (1.2 mL) and stirred at 80 ^0^C under argon for 2 h. The reaction mixture was cooled, diluted with water (30 mL), extracted with EtOAc (3 x 20 mL) and concentrated. The residue was purified by silica gel column chromatography using EtOAc/hexanes (05: 95) to isolate the product *tert*-butyl-2-(*tert*-butoxycarbonyl((5-((trimethylsilyl)ethynyl)pyridine-2-yl)methyl)amino)ethanoate as a colorless oil (1.022 g, 98%). ^1^H NMR (300 MHz, CDCl_3_) δ 8.59-8.57 (m, 1H, Py), 7.74-7.69 (m, 1H, Py), 7.35-7.25 (m, 1H, Py), 4.61 (s, 1H, PyCH_2_), 4.55 (s, 1H, PyCH_2_), 3.98 (s, 1H, NCH_2_), 3.82 (s, 1H, NCH_2_), 1.46-1.39 (m, 18H, CH_3_), 0.25 (s, 9H, SiCH_3_); ^13^C NMR (75 MHz, CDCl_3_) δ 168.7(CO), 168.6(CO), 157.6(CO), 157.1(CO), 155.4(Py), 155.4(Py), 151.8(Py), 151.7(Py), 139.4(Py), 139.3(Py), 121.5(Py), 120.3(Py), 118.7(Py), 118.5(Py), 101.4(alkyne), 101.3(alkyne), 97.8(alkyne), 81.4(O-C), 81.3(O-C), 80.4(O-C), 80.3(O-C), 53.7(NCH_2_CO), 53.2(NCH_2_CO), 49.9(PyCH_2_), 49.8(PyCH_2_), 28.1(CH_3_), 27.9(CH_3_), 25.0(CH_3_), -0.36(SiCH_3_); IR (Neat): 2158(alkyne), 1743(CO), 1701(CO) cm^-1^. Silver triflate (0.981g, 0.38 mmol, 20 mol%) was added to the *tert*-butyl 2-(*tert*-butoxycarbonyl((5-((trimethylsilyl)ethynyl)pyridin-2-yl)methyl)amino)acetate (0.800 g, 1.91 mmol) in a mixture of CH_2_Cl_2_/MeOH/H_2_O (7: 4: 1; 12 mL) and allowed to stir at room temperature for 20 h. Saturated NaHCO_3_ (30 mL) was added and the product was extracted with dichloromethane (60 mL). The residue was purified by silica gel column chromatography using EtOAc/hexanes (10: 90) to isolate *tert*-butyl-2-(*tert*-butoxycarbonyl((5-ethynylpyridine-2-yl)methyl)amino)ethanoate (0.600 g, 90%) as a colorless solid. ^1^H NMR (300 MHz, CDCl_3_, mixture of carbamate rotamers observed) δ 8.63-8.60 (m, 1H, Py), 7.78-7.73 (m, 1H, Py), 7.40-7.30 (m, 1H, Py), 4.62 (s, 1H, PyCH_2_), 4.57 (s, 1H, PyCH_2_), 3.99 (s, 1H, NCH_2_), 3.86 (s, 1H, NCH_2_), 3.29-3.27 (m, 1H, alkyne), 1.48-1.41 (m, 18H, CH_3_); ^13^C NMR (75 MHz, CDCl_3_) δ 168.5(CO), 168.4(CO), 157.8(CO), 157.4(CO), 155.2(Py), 155.1(Py), 151.8(Py), 151.6(Py), 139.4(Py), 139.3(Py), 121.4(Py), 120.4(Py), 117.5(Py), 117.4(Py), 81.2 (alkyne), 81.0(alkyne), 80.4(O-C), 80.4(O-C), 80.2(O-C), 80.1(O-C), 80.0(O-C), 80.0(O-C), 77.2(O-C), 53.5(NCH_2_), 53.1(NCH_2_), 49.9(PyCH_2_), 49.6(PyCH_2_), 27.9(CH_3_), 27.7(CH_3_), 27.7(CH_3_); IR (KBr): 2106 (alkyne), 1742(CO), 1687(CO) cm^-1^.

Iodide **1** (0.248 g, 0.5 mmol), alkyne (0.190 g, 0.547 mmol), PdCl_2_(PPh_3_)_2_  (0.035 g, 0.05 mmol, 10 mol%) and copper iodide (0.009 g, 0.05 mmol, 10 mol%) weighed into a 25 mL round bottomed flask. NMP (3 mL) and dry Et_3_N (0.5 mL) was added to it, and allowed to stir at room temperature under argon for 6 h. The reaction mixture was cooled and diluted with de-ionized water (50 mL) and the product was extracted with ethyl acetate (80 mL), evaporated under vacuum and dried. The residue was purified by silica gel column chromatography using EtOAc/hexanes (10: 90) to isolate the product G-CC-5-pyridyl-2-CH_2_-N(*^t^*Boc)CH_2_CO_2_*^t^*Bu (0.321 g, 90%) as a pale yellow solid. ^1^H NMR (300 MHz, CDCl_3_, mixture of carbamate rotamers observed) δ 8.64-8.61 (m, 1H, Ar), 7.76-7.72 (m, 1H, Ar), 7.35 (d, *J* = 8.2 Hz, 1H, Ar ), 7.29-7.26 (m, 1H, Ar), 7.16 (dd, *J* = 8.2, 2.0 Hz, 1H, Ar), 7.13 (s, 1H, Ar), 7.03 (s, 1H, Ar), 6.58 (d, *J* = 8.2, 1H, Ar), 6.00 (d, *J* = 1.5 Hz, 1H, OCH_2_O), 5.99 (d, *J* = 1.5 Hz, 1H, OCH_2_O), 5.92-5.88 (m, 1H, H-1), 5.71-5.66 (m, 1H, H-2), 4.93 (d, *J* = 3.0 Hz, 1H, H-4), 4.62 (s, 1H), 4.56 (s, 1H), 4.08 (d, *J* = 9.0 Hz, 1H, H-9b) 3.99 (s, 1H, NCH_2_), 3.84 (s, 1H, NCH_2_), 3.76 (bs, 1H, NH), 3.23-3.11 (m, 1H, H-3), 2.59-2.50 (m, 1H, H-3), 1.85-1.79 (m, 1H, H-3a), 1.47-1.41 (m, 18H, CH_3_); ^13^C NMR (75 MHz, CDCl_3_) δ 168.7(CO), 168.6(CO), 156.5(CO), 155.1(CO), 155.4(CO), 155.4(CO), 151.1(Py), 151.0(Py), 147.44(Ar), 146.0(Ar), 138.7(Py), 138.5(Py), 133.8(C-1), 133.6(Ar), 132.5(Ar), 130.3, 129.8(C-2), 125.8(Ar), 121.6(Ar), 120.5(Ar), 119.5(Ar), 119.3(Ar), 115.8(Ar), 112.9(Ar), 112.7(Ar), 112.3(Ar), 112.3(Ar), 107.7(Ar), 101.6(OCH_2_O), 93.4(alkyne), 83.9(alkyne), 83.8(alkyne), 81.4(O-C), 81.2(O-C), 80.4(O-C), 80.3(O-C), 77.2(O-C), 56.2(C-3a), 53.6(C-9b), 53.2(NCH_2_CO), 49.9(PyCH_2_N), 49.7(PyCH_2_N), 45.4(C-3), 41.9(C-3), 31.2(C-3a), 28.1(CH_3_), 27.9(CH_3_); IR (KBr): 2205(alkyne), 1744(CO), 1700(CO) cm^-1^.

3. Synthesis of chemical intermediates for ligand **18** (Refer to Figures S6 and S7).

Bromide **11** (0.753 g, 1.50 mmol), 4-ethynylaniline (0.263 g, 2.25 mmol), Pd(OAc)_2_ (0.034 g, 0.15 mmol, 10 mol%), triphenylphosphine (0.078 g, 0.30 mmol, 20 mol%) and CuI (0.057 0.30 mmol, 20 mol%) weighed into a 25 mL round bottom flask under argon. Dry diethylamine (2 mL) was added, and the reaction mixture was heated at 55 °C under argon for 2 h. The volatiles were removed in vacuo, and the residue was purified by silica gel column chromatography using EtOAc/hexanes (25: 75) to isolate the product di-*tert*-butyl 1-(5-2(4-aminophenyl)ethynyl)pyridin-2-yl)-2-(2-tert-butoxy-2-oxoethyl)hydrazine-1, 2-dicarboxlate (0.550 g, 68%) as pale yellow solid. ^1^H NMR (300 MHz, CDCl_3_) δ 8.46-8.44 (m, 1H, Py), 7.75-7.68 (m, 1H, Py), 7.59-7.55 (m, 1H, Py), 7.32-7.28 (m, 2H, Ar), 6.60-6.55 (m, 2H, Ar), 4.34-4.27 (m, 1H, NCH_2_CO), 4.11-4.05 (m, 1H, NCH_2_CO), 4.01 (bs, 2H, NH_2_), 1.53-1.39 (m, 27H, CH_3_); ^13^C NMR (75 MHz, CDCl_3_) δ 167.1(CO), 167.0(CO), 154.5(CO), 154.0(CO), 152.5(CO), 152.2(CO), 151.5(Py), 151.1(Py), 149.6(Ar), 147.2(Ar), 139.5(Py), 139.2(Py), 132.7(Py), 118.0(Py), 117.6(Py), 117.5(Py), 116.7(Py), 114.4(Py), 111.1(Ar), 111.0(Ar), 93.0(alkyne), 83.5(alkyne), 83.5(O-C), 82.6(O-C), 82.0(O-C), 81.0(O-C), 81.0(O-C), 77.2(O-C), 54.7(NCH_2_CO), 52.9(NCH_2_CO), 27.9(CH_3_), 27.9(CH_3_), 27.7(CH_3_); IR (KBr): 3377(NH), 2212(Alkyne), 1724(CO) cm^-1^. Di-*tert*-butyl 1-(5-2(4-aminophenyl)ethynyl)pyridin-2-yl)-2-(2-*tert*-butoxy-2-oxoethyl)hydrazine-1, 2-dicarboxlate (0.538 g, 1 mmol) and Pd/C (10%) (0.540 g) were combined in a Parr hydrogenation flask, ethanol (20 mL) was added, the apparatus was pressurized with hydrogen (30 psi) and allowed to shake for 3 h. The reaction mixture was filtered through Fluorosil, the filtrate was evaporated under reduced pressure and the residue was purified by silica gel column chromatography using EtOAc/hexanes (25: 75) to isolate the product **17** (0.490 g, 91%) as a viscous oil. ^1^H NMR (300 MHz, CDCl_3_) δ 8.16 (m, 1H, Py), 7.51-7.43 (m, 2H, Py), 6.91 (d, *J* = 8.2 Hz, 2H, Ar), 6.57 (d, *J* = 8.2 Hz, 2H, Ar), 4.40-4.31 (m, 1H, NCH_2_CO), 4.06-4.00 (m, 1H, NCH_2_CO), 3.49 (bs, 2H, NH_2_), 2.86-2.78 (m, 4H, ArCH_2_CH_2_Py), 1.51-1.36 (m, 27H, CH_3_); ^13^C NMR (75 MHz, CDCl_3_) δ 167.2(CH_2_CO), 167.1(CH_2_CO), 154.5(CO), 153.9 (CO), 152.7(CO), 152.4(CO), 151.3(CO), 150.9(Py), 147.3(Py), 144.5(Ar), 137.5(Py), 137.2(Py), 134.6(Ar), 134.3(Py), 130.3(Py), 130.2(Py), 128.8(Ar), 118.6(Py), 117.6(Py), 114.9(Ar), 81.9(O-C), 81.8(O-C), 81.1(O-C), 77.1(O-C), 54.7(NCH_2_CO), 53.0(NCH_2_CO), 36.5(CH_2_), 34.4(CH_2_), 34.3(CH_2_), 27.8(CH_3_), 27.6(CH_3_); IR (KBr): 3435(NH), 1725 (CO)cm^-1^.

The amine **17** (0.480 g, 0.88 mmol) and 6-bromopiperonal (0.202 g, 0.88 mmol) were refluxed in benzene for 30 h. Volatiles were evaporated under reduced pressure. The intermediate imine was dissolved in MeOH (5 mL), KHSO_4_ (0.120 g, 0.88 mmol) was added, followed by cyclopentadiene (1 mL), and the reaction mixture was stirred at rt for 10 h. The volatiles were removed *in vacuo* and the residue was purified by silica gel column chromatography using EtOAc/hexanes (15: 85) to isolate the product G-CH_2_CH_2_-5-pyridyl-2-N(*^t^*Boc)-N(*^t^*Boc)CH_2_CO_2_*^t^*Bu (0.302 g, 42%) as a pale yellow solid, consisting of a mixture of syn: anti (3.3:1) determined by NMR; ^1^H NMR (300 MHz, CDCl_3_) δ 8.21-8.19 (m, 1H, Py), 7.54-7.42 (m, 2H, Py and Ar), 7.17 (s, 1H, Ar), 6.87 (d, *J* = 2.0 Hz, 1H, Ar), 6.79 (d, *J* = 8.5 Hz, 1H, Ar), 6.55 (d, *J* = 8.5 Hz, 1H, Ar), 5.98 (d, *J* = 1.5 Hz, 1H, OCH_2_O), 5.97 (d, *J* = 1.5 Hz, 1H, OCH_2_O), 5.87-5.80 (m, 1H, H-1), 5.67-5.62 (m, 1H, H-2), 4.86 ( d, *J* = 3.0 Hz, 1H, H-4), 4.41-4.25 (m, 2H, H-9b and NCH_2_CO), 4.10-3.99 ( m, 2H, H-9b and NCH_2_CO), 3.50 (bs, 1H, NH), 3.22-3.11 (m, 1H, H-3), 2.88-2.74 (m, 4H, CH_2_), 2.63-2.53 (m, 1H, H-3), 1.83-1.75 (m, 1H, H-3a), 1.52-1.36 (m, 27H, CH_3_); ^13^C NMR (75 MHz, CDCl_3_) δ 167.4(CO), 167.3(CO), 154.7(CO), 154.1(CO), 152.9(CO), 152.6(CO), 151.5(Py), 151.1(Py), 147.8(Py), 147.5(Ar), 147.4(Ar), 147.1(Py), 143.4(Py), 137.7(Py), 137.7(Py), 137.4(Py), 134.8(Py), 134.5(C-1), 133.9(Ar), 133.9(Ar), 131.8(Ar), 131.7(Ar), 131.7(Ar), 130.2(Ar), 130.2(C-2), 128.7(Ar), 126.2(Ar), 126.0(Ar), 118.6(Ar), 117.8(Py), 116.1(Ar), 112.9(Ar), 112.7(Ar), 108.0(Ar), 101.6(OCH_2_O), 82.1(O-C), 81.8(O-C), 81.4(O-C), 80.9(O-C), 77.2(O-C), 56.7(C-4), 54.9(N-CH_2_-CO), 53.2(N-CH_2_-CO), 46.0(C-9b), 41.0 (C-3), 36.7(CH_2_), 34.6(CH_2_), 34.5(CH), 31.2(C-3a), 28.1(CH_3_), 28.0(CH_3_), 28.0(CH_3_), 27.8(CH_3_); IR (KBr): 3442(NH),1720(CO) cm^-1^.

4. Synthesis of chemical intermediates for ligand **20** (Refer to Figures S8 and S9).

Bromide **11** (0.765 g, 1.52 mmol), bis-pinacolatodiboron (0.578 g, 2.29 mmol), PdCl_2_dppf·CH_2_Cl_2_ (0.099 g, 0.122 mmol, 8 mol %), and KOAc (0.744 g, 7.6 mmol) were combined in a Schlenk tube. Dry DMF (5 mL) was added and the reaction was heated at 80 °C for 16 h. The reaction mixture was diluted with hexane and washed with saturated NH_4_Cl, water, and brine solution. The organic layer was dried over Na_2_SO_4_, filtered, and concentrated. The crude residue was purified by column chromatography using hexanes to isolate di-*tert*-butyl 1-(2-*tert*-butoxy-2-oxoethyl)-2-(5-(4, 4, 5, 5-tetramethyl-1, 3, 2-dioxaborolan-2-yl)pyridin-2-yl)hydrazin-1,2-dicarboxylate (0.785 g, 94%) as a colorless solid. ^1^H NMR (300 MHz, CDCl_3_) δ 8.70 (m, 1H, Py), 8.03 (m, 1H, Py), 7.60 (m, 1H, Py), 7.34 (s, 1H), 4.26-4.06 (m, 2H), 1.53-1.24 (m, 39H). ^13^C NMR (75 MHz, CDCl_3_) δ 167.2(CH_2_CO), 167.1(CH_2_CO), 155.2(CO), 154.8(CO), 154.7(CO), 154.0(CO), 153.9(Py), 153.8(Py), 152.6(Py), 143.7(Py), 143.4(Py), 117.2(Py), 116.3(Py), 83.9(O-C), 83.8(O-C), 83.3(O-C), 82.4(O-C), 81.8(O-C), 81.3(O-C), 80.9(O-C), 80.8(O-C), 54.8(N-CH_2_CO), 52.9(N-CH_2_CO), 28.0(CH_3_), 28.0(CH_3_), 27.9(CH_3_), 27.8(CH_3_), 27.8(CH_3_), 24.8(CH_3_), 24.7(CH_3_), 24.6(CH_3_), 24.4(CH_3_); IR (Neat): 1755(CO), 1723(CO) cm^-1^. Di-*tert*-butyl 1-(2-*tert*-butoxy-2-oxoethyl)-2-(5-(4,4,5,5-tetramethyl-1,3,2-dioxaborolan-2-yl)pyridin-2-yl)hydrazine-1,2-dicarboxylate (0.519 g, 0.945 mmol), *p*-iodoaniline (0.186 g, 0.85 mmol), KOH (0.095 g, 1.7 mmol), and palladacycle (C_14_H_12_N_2_Cl_2_O_4_Pd_2_)^7^ (10 mg, 17 mmol, 4 mol % Pd) were combined with methanol/water (4 mL, 3:1) and the reaction mixture was heated at 60 °C for 3 h. The mixture was diluted with ethyl acetate (5 mL) and washed with water (10 mL). The organic layer was dried over Na_2_SO_4_, filtered, and concentrated. The residue was purified by silica gel column chromatography using EtOAc/hexanes (30: 70) to isolate the amine product **19** (0.338 g, 70%) as a colorless solid. ^1^H NMR (300 MHz, CDCl_3_) δ 8.55-8.53 (m, 1H, Py), 7.82-7.78 (m, 1H, Py), 7.62-7.53 (m, 1H, Py), 7.38-7.33 (m, 2H, Ar), 6.78-6.74 (m, 2H, Ar), 4.42-4.33 (m, 1H, NCH_2_CO), 4.08-4.01 (m, 1H, NCH_2_CO), 3.77 (s, 2H, NH_2_), 1.59-1.24 (m, 27H, CH_3_); ^13^C NMR (75 MHz, CDCl_3_) δ 167.4(CO), 167.3(CO), 154.1(CO), 152.9(CO), 152.6(CO), 151.5(CO), 151.1(Py), 146.5(Py), 145.2(Ar), 145.1(Py), 135.2(Py), 134.9(Py), 134.3(Py), 133.9(Py), 127.7(Ar), 127.7(Ar), 127.2(Py), 127.1(Ar), 118.8(Ar), 117.9(Py), 115.3(Py), 82.3(O-C), 81.9(O-C), 81.5(O-C), 81.0(O-C), 54.9(NCH_2_CO), 53.2(NCH_2_CO), 28.0(CH_3_), 27.8(CH_3_), 24.7(CH_3_); IR (Neat): 3467(NH), 1722 (CO), 1626 (CO)cm^-1^.

A neat mixture of aniline **19** (0.208 g, 0.393 mmol) and 6-bromopiperonal was heated at 180 °C for 5 min to generate the intermediate imine, then dissolved in methanol (2 mL), cyclopentadiene (1 mL) and KHSO_4_ (0.022 g, 0.157 mmol) were added, and the reaction mixture was stirred at room temperature overnight. The reaction mixture was concentrated, and the residue was purified by silica gel column chromatography using EtOAc/hexanes (40: 60) to isolate the product G-5-pyridyl-2-N(*^t^*Boc) N(*^t^*Boc)CH_2_CO_2_*^t^*Bu (0.224 g, 74%) as a colorless solid, consisting of a mixture of syn: anti (3: 1). determined by NMR; ^1^H NMR (300 MHz, CD_3_OD): δ 8.51-8.49 (m, 1H, Py), 7.98-7.92 (m, 1H, Py), 7.61-7.54 (m, 1H, Ar), 7.29 (m, 1H, Ar), 7.22-7.18 (m, 1H, Ar), 7.16 (s, 1H, Ar), 7.03 (s, 1H, Ar), 6.77 (dd, *J* = 8.3, 1.4 Hz, 1H, Ar), 5.97 (d, *J* = 1.0 Hz, 1H, OCH_2_O), 5.95 (d, *J* = 1.0 Hz, 1H, OCH_2_O), 5.93-5.90 (m, 1H, H-1), 5.62-5.60 (m, 1H, H-2), 4.82 (bs, 1H, H-4), 4.39-4.32 (m, 1H, NCH_2_CO), 4.09-3.99 (m, 2H, NCH_2_CO and H-9b), 3.17-3.13(m, 1H, H-3), 2.55-2.48 (m, 1H, H-3), 1.75-1.65 (m, 1H, H-3a), 1.51-1.32 (m, 27H, CH_3_); ^13^C NMR (75 MHz, CDCl_3_) δ 167.4(CO), 167.3(CO), 154.7(CO), 154.1(CO), 152.9(CO), 152.6(CO), 151.6(Py), 151.2(Py), 147.4(Ar), 147.2(Ar), 145.3, 145.3, 135.3(C-1), 135.0(Ar), 134.3(Ar), 134.1(Ar), 134.12(Ar), 134.0(Ar), 133.9(Ar), 133.7(Ar), 130.5(C-2), 128.5(Ar), 128.6(Ar), 127.5(Ar), 127.4(Ar), 126.5(Ar), 126.5(Ar), 124.9(Ar), 124.9(Ar), 118.7(Ar), 117.8(Ar), 117.8(Ar), 116.6(Ar), 112.9(Py), 112.8(Py), 107.9(Ar), 101.7(Ar), 82.3(O-C), 81.4(O-C), 81.0(O-C), 56.5 (C-4), 54.9(N-CH_2_-CO), 53.24(C-9b), 42.1(C-3), 31.2(C-3a), 28.1(CH_3_), 28.0(CH_3_), 27.8(CH_3_); IR (KBr): 3435(NH), 1722(CO) cm^-1^.

5. Synthesis of chemical intermediates for ligand **23, 25** (Refer to Figures S10, S11 and S12).

A catalytic amount of Sc(OTf)_3_ (0.025 g, 10 mol %) in anhydrous acetonitrile (0.5 mL) was added to the mixture of 6-bromopiperonal (0.115 g, 0.5 mmol), 4-azidoaniline **21** (0.067 g, 0.5 mmol), and cyclopentadiene (0.163 g, 2.5 mmol) in acetonitrile (3 mL). The reaction mixture was stirred at ambient temperature (~23˚C) for 6 h with monitoring the product formation by thin layer chromatography using ethyl acetate/hexanes as eluent. The volatiles were removed *in vacuo*. The residue was purified by silica gel column chromatography using EtOAc/hexanes (3: 97) to isolate the azide product **22** (0.185 g, 90%) as a yellow solid consisting of a mixture of syn: anti (11.5: 1) determined by NMR; ^1^H NMR (300 MHz, CDCl_3_) δ 7.15 (s, 1H, Ar), 7.03 (s, 1H, Ar), 6.72-6.59 (m, 3H, Ar), 6.00 (m, 2H, OCH_2_O), 5.85-5.82 (m, 1H, H-1), 5.70-5.64 (m, 1H, H-2), 4.85 (d, *J* = 2.9 Hz, 1H, H-4), 4.07 (d, *J* = 8.5 Hz, 1H, H-9b), 3.55 (bs, 1H, NH), 3.20-3.15 (m, 1H, H-3), 2.59-2.53 (m, 1H, H-3), 1.84-1.78 (m, 1H, H-3a); ^13^C NMR (50 MHz, CDCl_3_) δ 147.5(Ar), 147.3(Ar), 142.7(Ar), 134.1(C-1), 133.4(Ar), 130.7(Ar), 130.6(C-2), 127.6(Ar), 119.3(Ar), 117.2(Ar), 113.0(Ar), 112.8(Ar), 107.9(Ar), 101.7(OCH_2_O), 56.7(C-4), 46.0(C-9b), 41.9(C-3), 31.3(C-3a); IR (KBr): 3357(NH), 2109(alkyne) cm^-1^; HPLC-MS: Eluting with 60-90% CH_3_CN(gradient 1 % min^-1^) in H_2_O, exhibited single peak at R_t_ = 19.03 min. ESI-MS *m/z* (ES+) calcd [M – N_2_] ^+^ for C_19_H_15_BrN_4_O_2_ 383.04, found 383.04.

Azide **22** (0.123 g, 0.3 mmol), Alkyne **12** (0.134 g, 0.3 mmol), CuSO_4_.5H_2_O (0.007g, 10 mol%), and sodium ascorbate (0.012 g, 20 mol%) were dissolved in water/^t^BuOH (2: 2 mL) and heated at 60 °C for 20 h. The reaction was monitored by TLC using ethyl acetate/hexanes (30: 70) as eluent. Water (30 mL) was added to the reaction mixture and then extracted with ethyl acetate (60 mL). The organic layer was evaporated under reduced pressure. The residue was purified by silica gel column chromatography using EtOAc/hexanes (25: 75) to isolate the product G-triazole-5-pyridyl-2-N(*^t^*Boc) N(*^t^*Boc)CH_2_CO_2_*^t^*Bu as a yellowish solid (0.134 g, 52%). ^1^H NMR (300 MHz, CDCl_3_, mixture of carbamate rotamers observed) δ 8.86-8.83 (m, 1H, Tri), 8.27-8.19 (m, 1H, Py), 8.10-8.08 (m, 1H, Py), 7.73 (m, 1H, Py), 7.45-7.44 (m, 1H, Ar), 7.35-7.32 (m, 1H, Ar), 7.15 (s, 1H, Ar), 7.04 (m, 1H, Ar), 6.74 (dd, *J* = 8.5, 2.0 Hz, 1H, Ar), 6.01 (d, *J* = 1.1 Hz, 1H, OCH_2_O), 6.00 (d, *J* = 1.1 Hz, 1H, OCH_2_O), 5.92-5.91 (m, 1H, H-1), 5.75-5.71 (m, 1H, H-2), 4.95 (d, *J* = 2.9 Hz, 1H, H-4), 4.39-4.31 (m, 1H, NCH_2_CO), 4.17 (d, *J* = 9.0 Hz, 1H, H-9b), 4.12-4.04 (m, 1H, NCH_2_CO), 3.86 (bs, 1H, NH), 3.30-3.18 (m, 1H, H-3), 2.65-2.53 (m, 1H, H-3), 1.88-1.80 (m, 1H, H-3a), 1.55-1.39 (m, 27H, CH_3_); ^13^C NMR (50 MHz, CDCl_3_) δ 167.3(CO), 167.2(CO), 154.7(CO), 154.2(CO), 153.0(CO), 152.78(CO), 152.72(Py), 152.5(Py), 147.5(Py), 147.4(Tri), 146.2(Ar), 146.1(Py), 144.9(Py), 144.8(Tri), 144.7(Ar), 144.6(Ar), 134.8(C-1), 134.6(Ar), 133.76(Ar), 133.72(Ar), 133.3(Ar), 133.2(Ar), 131.0(Ar), 130.9(C-2), 128.93(Ar), 128.90(Ar), 127.08(Ar), 127.06(Ar), 124.0(Ar), 123.7(Ar), 121.7(Ar), 119.2(Ar), 119.1(Ar), 118.5(Ar), 117.8(Ar), 117.7(Ar), 117.6(Ar), 116.5(Ar), 113.0(Ar), 112.9(Ar), 107.8(Ar), 101.8(OCH_2_O), 82.67(O-C), 82.65(O-C), 82.1(O-C), 81.6(O-C), 81.1(O-C), 56.5(C-4), 55.0(NCH_2_CO), 53.2(NCH_2_CO), 45.8(C-9b), 41.9(C-3), 31.3(C-3a), 28.1(CH_3_), 28.0 (CH_3_), 27.95(CH_3_), 27.92(CH_3_); IR (KBr): 3423(NH), 1725(CO) cm^-1^.

Azide **22** (0.123 g, 0.3 mmol), alkyne **24** (0.050 g, 0.3 mmol), CuSO_4_.5H_2_O (0.007g, 10 mol %), and sodium ascorbate (0.012 g, 20 mol %) were combined in water/^t^BuOH (2 mL) (1: 1) and heated at 60 °C for 3 h. The progress of the reaction was monitored by TLC using MeOH/CH_2_Cl_2_ (5: 95) as eluent. Water (30 mL) was added to the reaction mixture and the product was extracted with ethyl acetate (60 mL) washed with 1M NH_4_OH solution (10 mL), dried over anhydrous sodium sulfate and concentrated in vacuo. The residue was purified by silica gel column chromatography using MeOH/CH_2_Cl_2_ (2: 95) to isolate the product G-triazole-CH_2_N(^t^Boc)CH_2_CO_2_*^t^*Bu (0.102 g, 60%). ^1^H NMR (300 MHz, CDCl_3_) δ 7.82 (s, 1H), 7.40 (d, *J* = 2.5 Hz, 1H), 7.27 (dd, *J* = 8.5, 2.5 Hz, 1H), 7.15 (s, 1H), 7.04 (s, 1H), 6.72 (d, *J* = 8.5 Hz, 1H), 6.01 (d, *J* = 1.4 Hz, 1H, OCH_2_O), 6.0 (d, *J* = 1.4 Hz, 1H, OCH_2_O), 5.91-5.87 (m, 1H, H-1), 5.72-5.69 (m, 1H, H-2), 4.94 (d, *J* = 3.0 Hz, 1H, H-4), 4.15 (d, *J* = 8.6 Hz, 1H, H-9b), 4.0 (s, 2H, NCH_2_CO), 3.82 (b s, 1H, NH), 3.40 (s, 2H, TriCH_2_N ), 3.25-3.17 (m, 1H, H-3), 2.62-2.53 (m, 1H, H-3), 2.00 (bs, 1H, NH), 1.86-1.79 (m, 1H, H-3a), 1.47 (s, 9H); ^13^C NMR (75 MHz, CDCl_3_) δ 171.3 (CO), 155.5(CO) 147.5(Ar), 147.4(Ar), 146.7(Ar), 145.8(Ar), 133.8(C-1), 133.3(Ar), 130.9(Tri), 129.2(C-2), 126.9(Ar), 121.6(Ar), 119.9(Ar), 119.0(Tri), 116.4(Ar), 113.0(Ar), 112.9(Ar), 107.8(Ar), 101.8(OCH_2_O), 81.3(OC), 56.5 (C-4), 50.9(NCH_2_CO), 45.8(C-9b), 44.2(TriCH_2_N), 41.9(C-3), 31.3(C-3a), 28.0(CH_3_); IR (KBr): 3353(NH),1731 (CO) cm^-1^.

References

1. Ramesh C, Bryant B, Nayak TK, Revankar CM, Anderson T et al. (2006) Linkage effects on binding affinity and activation of GPR30 and estrogen receptors ERalpha/beta with tridentate pyridin-2-yl hydrazine tricarbonyl-Re/(99m)Tc(I) chelates. *J. Am. Chem. Soc*. *128*: 14476-77.

2. Struthers H, Spingler B, Mindt TL, Schibli R, (2008) “Click-to-Chelate”: Design and Incorporation of Triazole-Containing Metal-Chelating Systems into Biomolecules of Diagnostic and Therapeutic Interest. *Chem. Eur. J*. *14*(20): 6173-83.

3. Ramesh C, Nayak TK, Burai R, Dennis MK, Hathaway HJ, et al. (2010) Synthesis and characterization of iodinated tetrahydroquinolines targeting the G protein-coupled estrogen receptor GPR30. *J. Med.* *Chem*. *53*: 1004-14.

# 4. Nishimura T, Maeda K, Ohsawa S, Yashima E (2005) Helical arrays of pendant fullerenes on optically active poly(phenylacetylene. Chem Eur. J. *11*(4): 1181-90.

5. Zhu W, Dawei MD (2004) Synthesis of aryl azides and vinyl azides *via* proline-promoted CuI-catalyzed coupling reactions. *Chem Commun*. (7): 888-9.

# 6. Kmentova I, Sutherland HS, Palmer BD, Blaser A, Franzblau SG, et al. (2010) Synthesis and structure−activity relationships of aza- and diazabiphenyl analogues of the antitubercular drug (6*S*)-2-nitro-6-{[4-(trifluoromethoxy)benzyl]oxy}-6,7-dihydro-5*H*-imidazo[2,1-*b*][1,3]oxazine (PA-824). *J. Med. Chem*, *53*: 8421-39.

# 7. Alonso DA, Najera C, Pacheco MC (2002) Highly active oxime-derived palladacycle complexes for Suzuki−Miyaura and Ullmann-type coupling reactions. *J. Org. Chem*. *67*: 5588-94.

**Figure S1** Synthetic scheme for preparation of 5-ethynyl derivative **12**.

(TIF)

**Figure S2** Synthetic scheme for preparation of G-CC-5-pyridyl-2-N(*^t^*Boc)N(*^t^*Boc)CH_2_CO_2_*^t^*Bu.

(TIF)

**Figure S3** Synthetic scheme for preparation of picoline amine derivative **15**.

(TIF)

**Figure S4** Synthetic scheme for preparation of *tert*-butyl-2-(*tert*-butoxycarbonyl((5-ethynylpyridine-2-yl)methyl)amino)ethanoate.

(TIF)

**Figure S5** Synthetic scheme for preparation of G-CC-5-pyridyl-2-CH_2_-N(*^t^*Boc)CH_2_CO_2_*^t^*Bu.

(TIF)

**Figure S6** Synthetic scheme for preparation of aniline derivative **17**.

(TIF)

**Figure S7** Synthetic scheme for preparation of G-CH_2_CH_2_-5-pyridyl-2-N(*^t^*Boc)-N(*^t^*Boc)CH_2_CO_2_*^t^*Bu.

(TIF)

**Figure S8** Synthetic scheme for preparation of aniline derivative **19**.

(TIF)

**Figure S9** Synthetic scheme for preparation of G-5-pyridyl-2-N(*^t^*Boc) N(*^t^*Boc)CH_2_CO_2_*^t^*Bu.

(TIF)

**Figure S10** Synthetic scheme for preparation of azide product **22**.

(TIF)

**Figure S11** Synthetic scheme for preparation of G-triazole-5-pyridyl-2-N(*^t^*Boc) N(*^t^*Boc)CH_2_CO_2_*^t^*Bu.

(TIF)

**Figure S12** Synthetic scheme for preparation of G-triazole-CH_2_N(^t^Boc)CH_2_CO_2_*^t^*Bu.

(TIF)
