# Supplementary figures and images for: Synthesis and Characterization of Tricarbonyl-Re/Tc(I) Chelate Probes Targeting the G Protein-Coupled Estrogen Receptor GPER/GPR30
Source: PLoS One. 2012 Oct 15;7(10):e46861. doi: 10.1371/journal.pone.0046861 (PMC3471960; doi:10.1371/journal.pone.0046861)

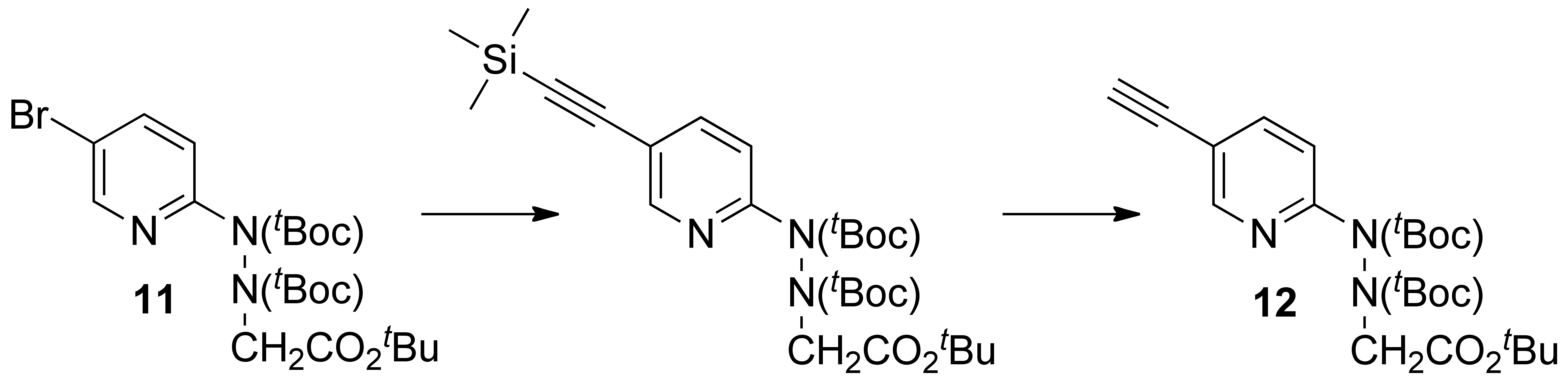

Supplement: Figure S1 — Synthetic scheme for preparation of 5-ethynyl derivative 12. (TIF) [file pone.0046861.s002.tif]

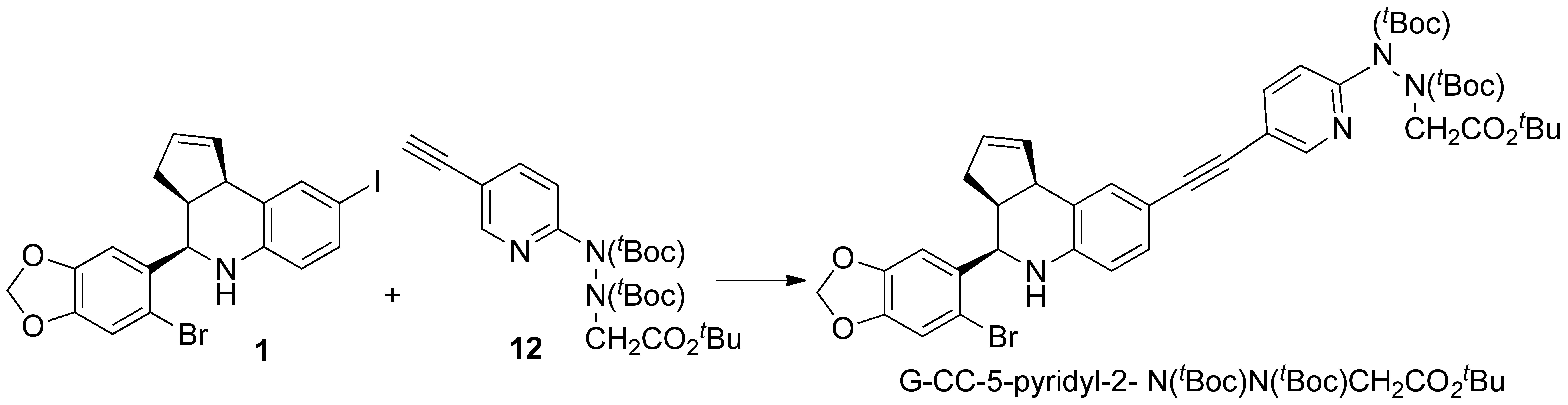

Supplement: Figure S2 — Synthetic scheme for preparation of G-CC-5-pyridyl-2-N( t Boc)N( t Boc)CH2CO2 t Bu. (TIF) [file pone.0046861.s003.tif]

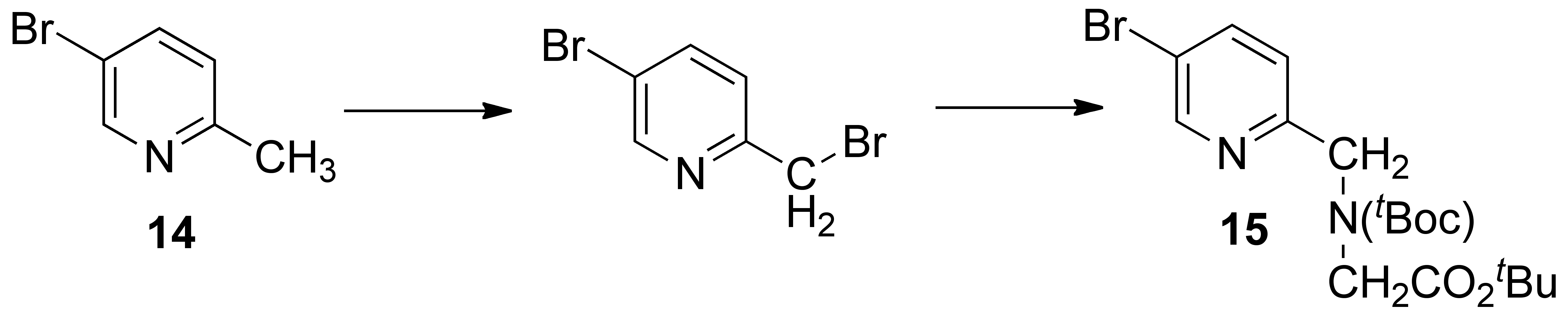

Supplement: Figure S3 — Synthetic scheme for preparation of picoline amine derivative 15. (TIF) [file pone.0046861.s004.tif]

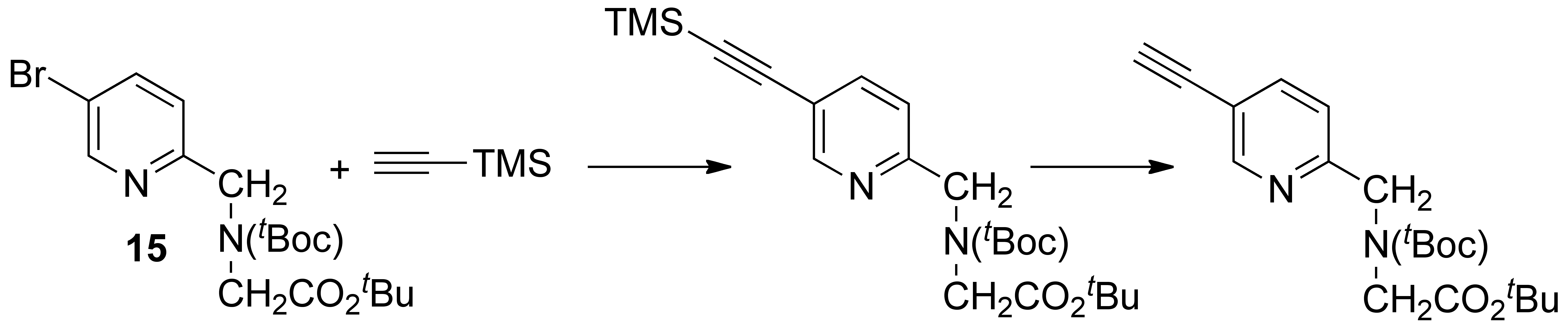

Supplement: Figure S4 — Synthetic scheme for preparation of tert -butyl-2-( tert -butoxycarbonyl((5-ethynylpyridine-2-yl)methyl)amino)ethanoate. (TIF) [file pone.0046861.s005.tif]

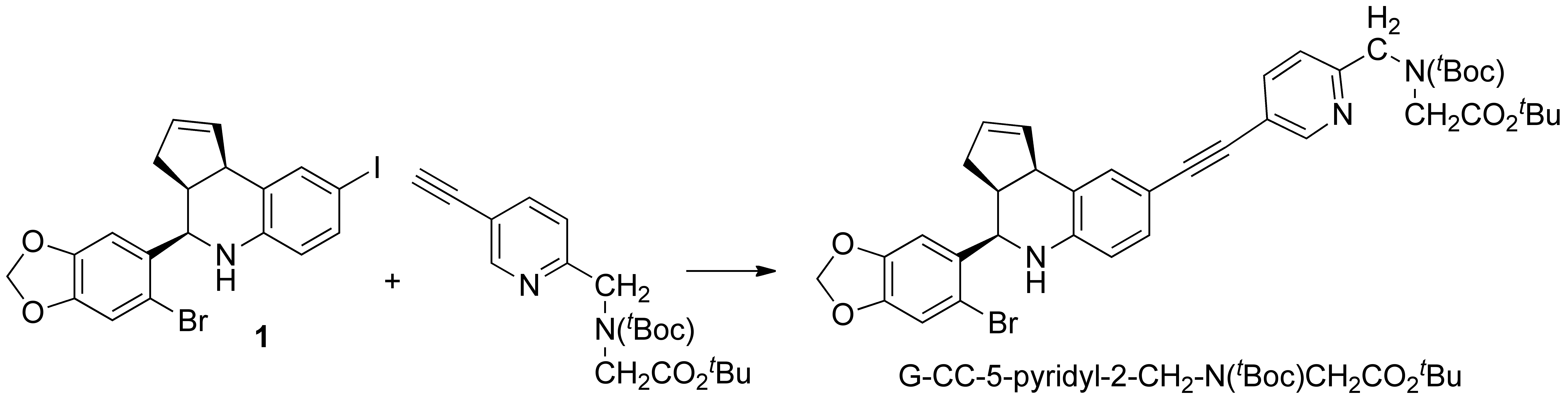

Supplement: Figure S5 — Synthetic scheme for preparation of G-CC-5-pyridyl-2-CH2-N( t Boc)CH2CO2 t Bu. (TIF) [file pone.0046861.s006.tif]

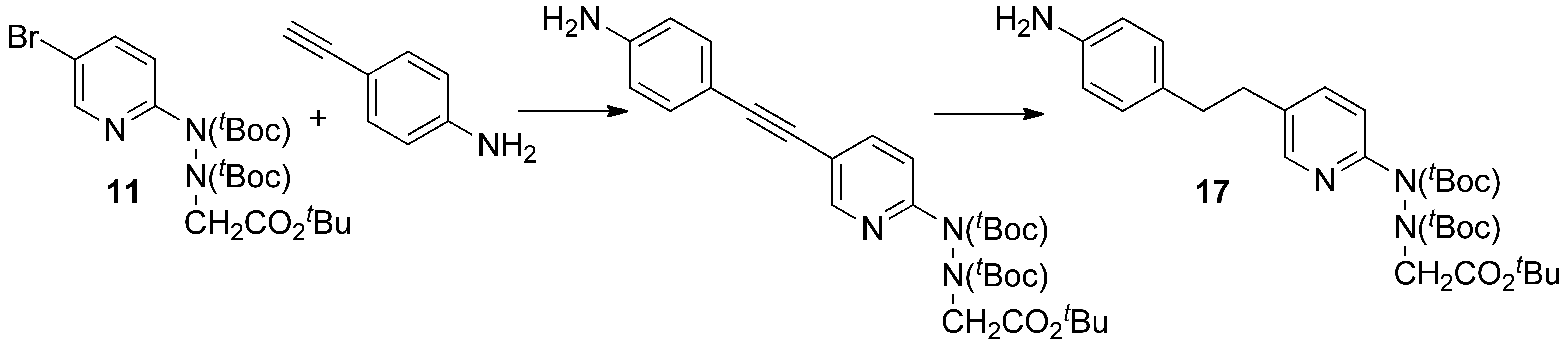

Supplement: Figure S6 — Synthetic scheme for preparation of aniline derivative 17. (TIF) [file pone.0046861.s007.tif]

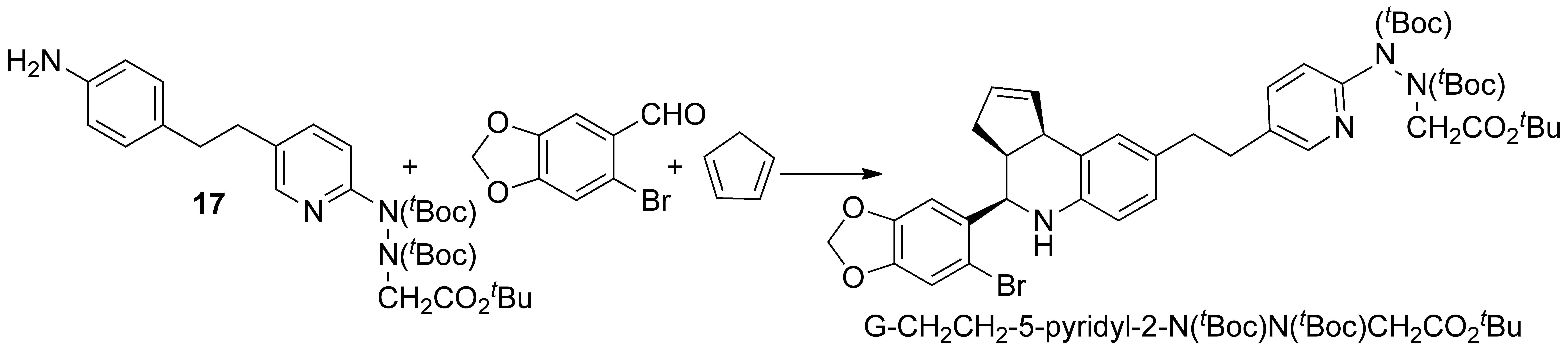

Supplement: Figure S7 — Synthetic scheme for preparation of G-CH2CH2-5-pyridyl-2-N( t Boc)-N( t Boc)CH2CO2 t Bu. (TIF) [file pone.0046861.s008.tif]

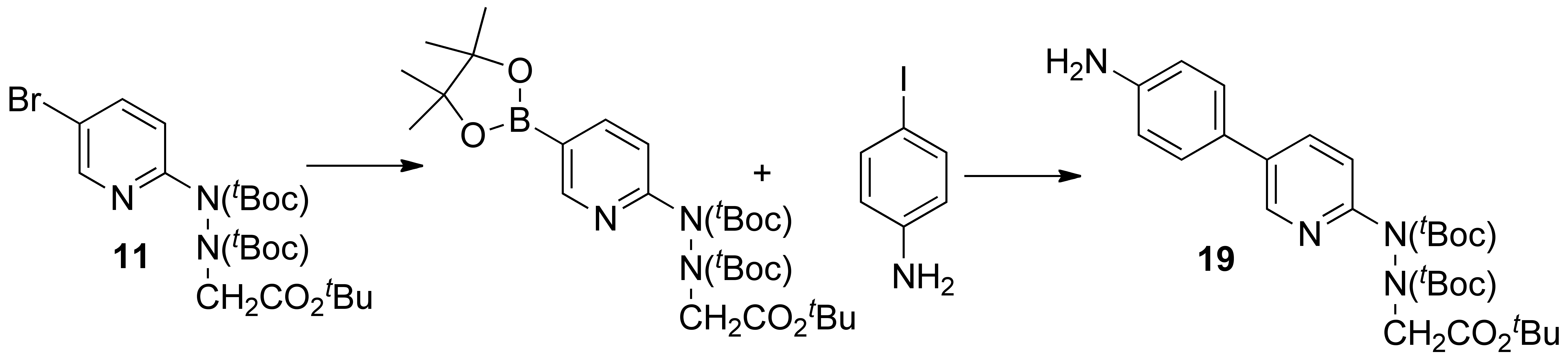

Supplement: Figure S8 — Synthetic scheme for preparation of aniline derivative 19. (TIF) [file pone.0046861.s009.tif]

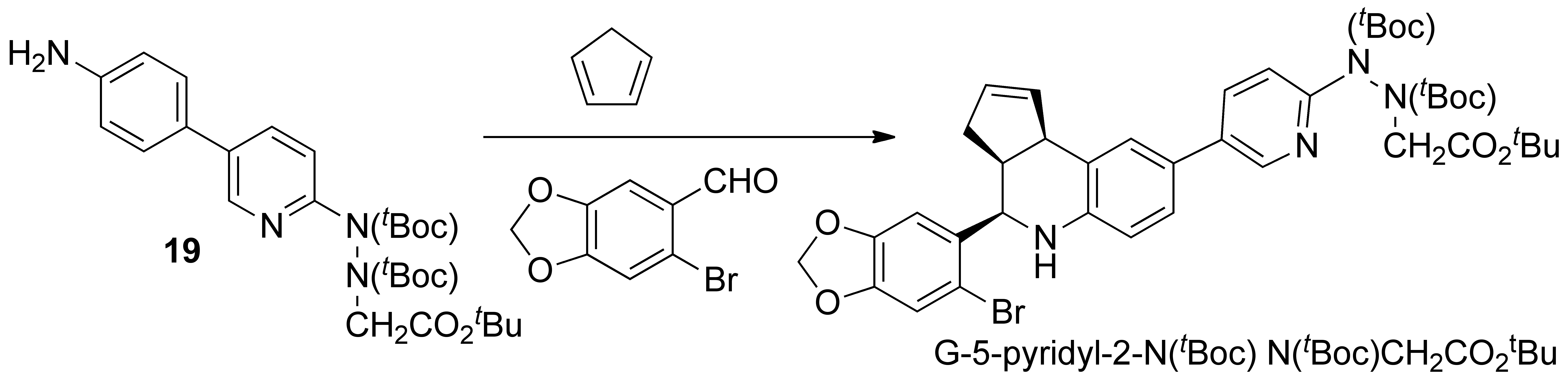

Supplement: Figure S9 — Synthetic scheme for preparation of G-5-pyridyl-2-N( t Boc) N( t Boc)CH2CO2 t Bu. (TIF) [file pone.0046861.s010.tif]

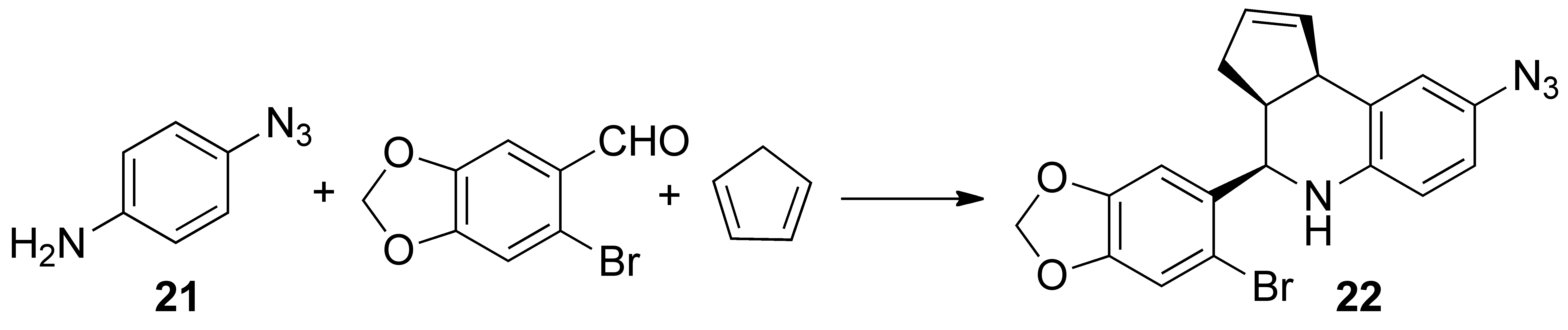

Supplement: Figure S10 — Synthetic scheme for preparation of azide product 22. (TIF) [file pone.0046861.s011.tif]

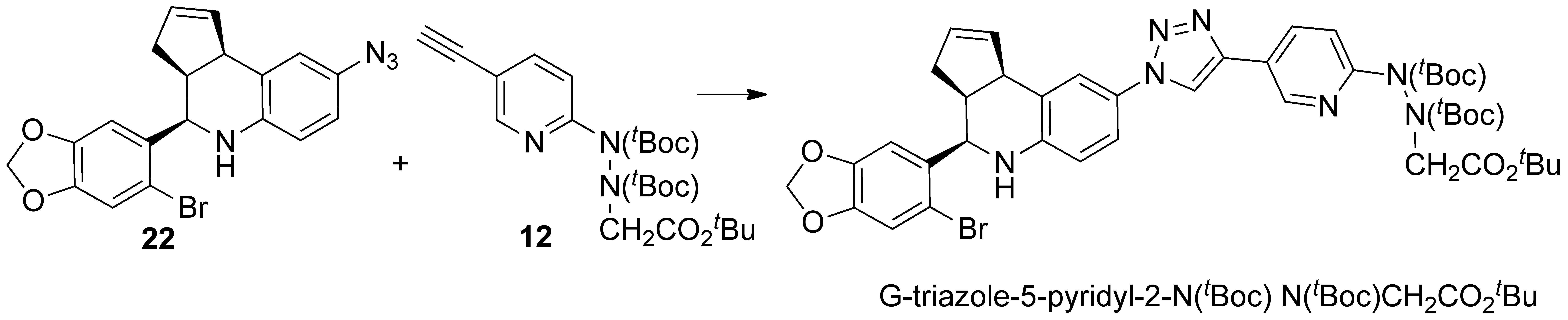

Supplement: Figure S11 — Synthetic scheme for preparation of G-triazole-5-pyridyl-2-N( t Boc) N( t Boc)CH2CO2 t Bu. (TIF) [file pone.0046861.s012.tif]

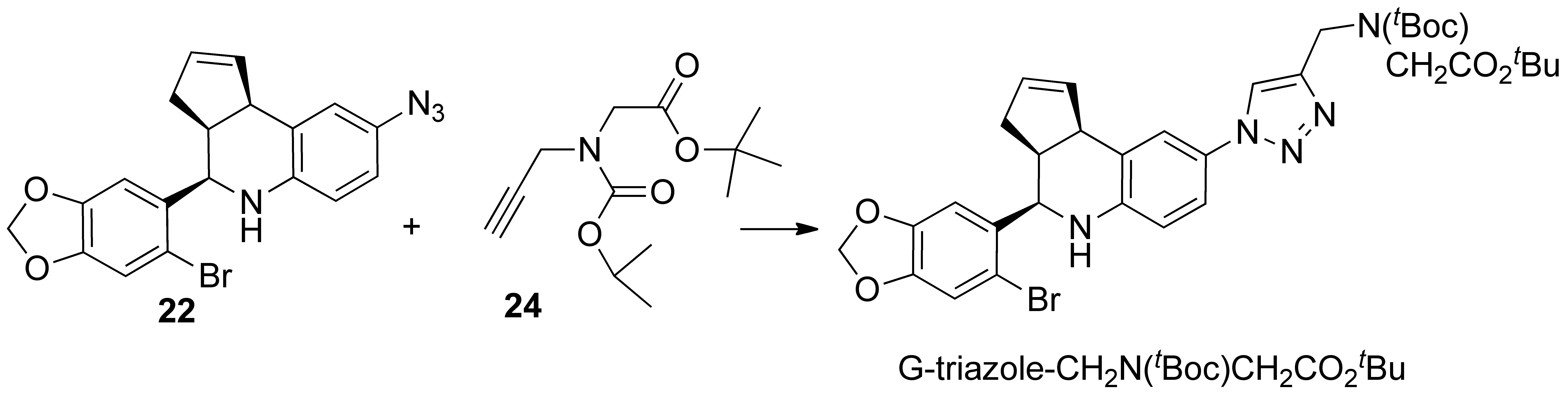

Supplement: Figure S12 — Synthetic scheme for preparation of G-triazole-CH2N(tBoc)CH2CO2 t Bu. (TIF) [file pone.0046861.s013.tif]
